# Supplementary material for: Competing Inter-Domain Quorum-Sensing Systems Control Prophage Lysis-Lysogeny Decisions
Source: bioRxiv. 2026 Jul 3:2026.07.02.736140. Preprint. [Version 1] doi: 10.64898/2026.07.02.736140 (PMC13345283; doi:10.64898/2026.07.02.736140)

bioRxiv preprint doi: https://doi.org/10.64898/2026.07.02.736140; this version posted July 3, 2026. The copyright holder for this preprint (which was not certified by peer review) is the author/funder, who has granted bioRxiv a license to display the preprint in perpetuity. It is made available under a CC-BY 4.0 International license.

Figure S1

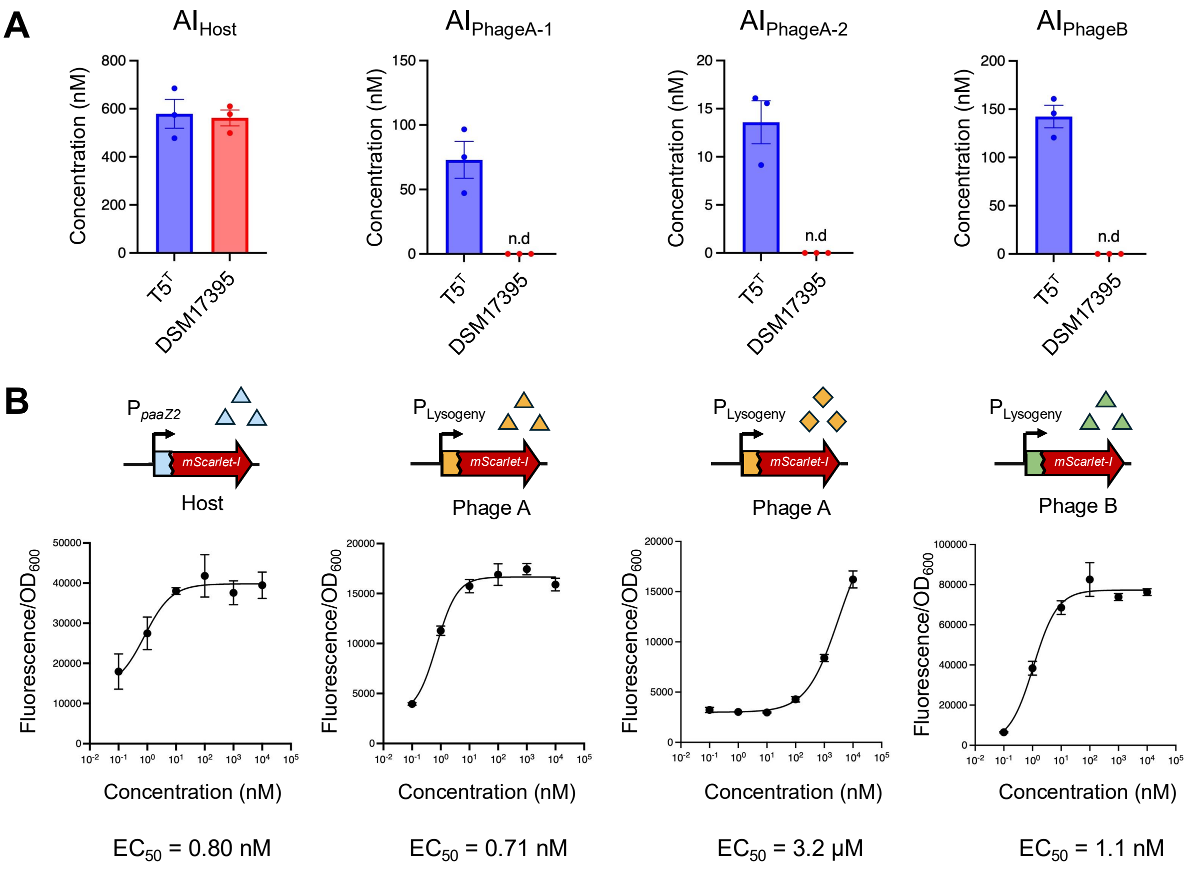

bioRxiv preprint doi: https://doi.org/10.64898/2026.07.02.736140; this version posted July 3, 2026. The copyright holder for this preprint (which was not certified by peer review) is the author/funder, who has granted bioRxiv a license to display the preprint in perpetuity. It is made available under a CC-BY 4.0 International license.

Figure S2

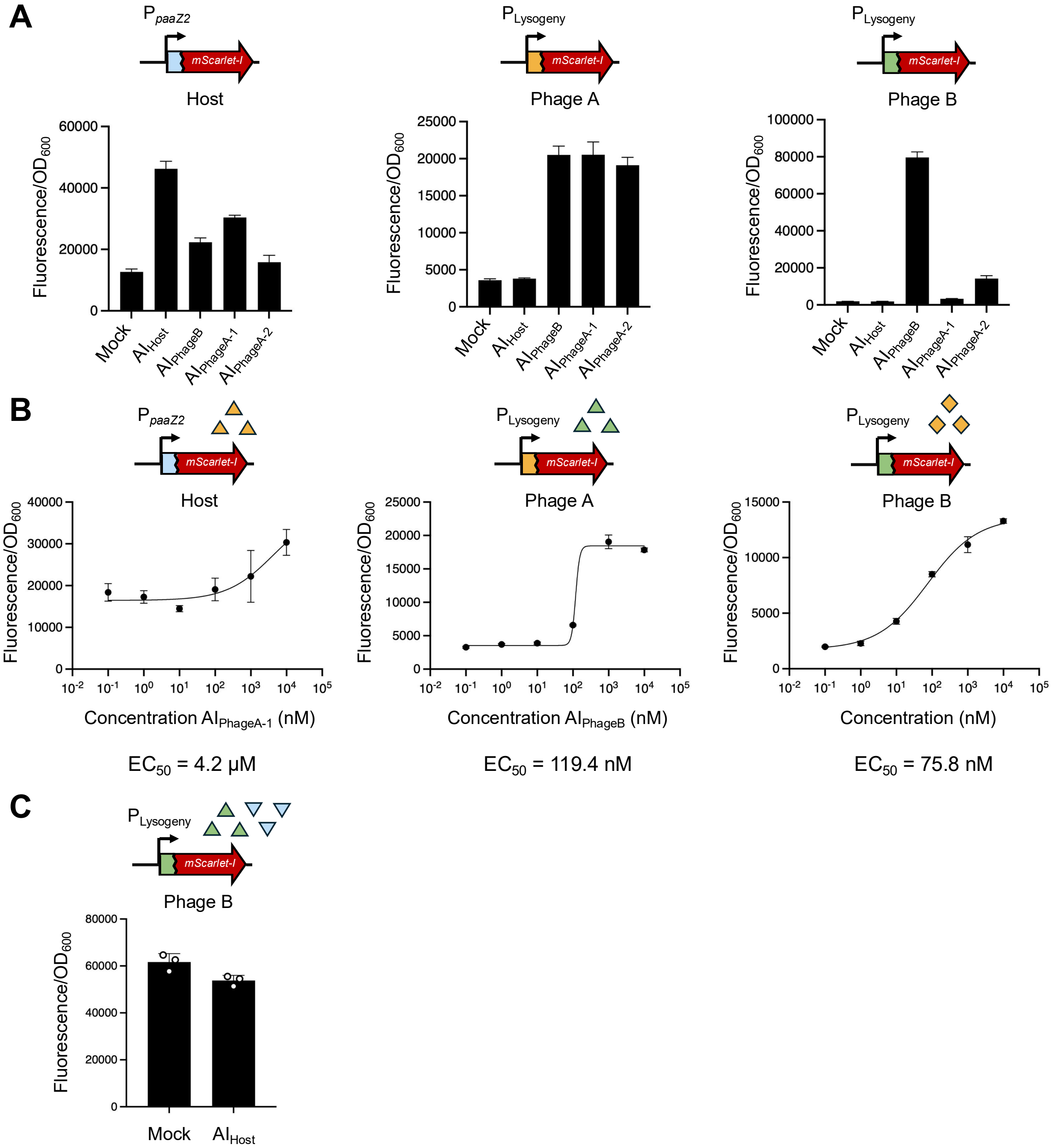

bioRxiv preprint doi: https://doi.org/10.64898/2026.07.02.736140; this version posted July 3, 2026. The copyright holder for this preprint (which was not certified by peer review) is the author/funder, who has granted bioRxiv a license to display the preprint in perpetuity. It is made available under a CC-BY 4.0 International license.

Figure S3

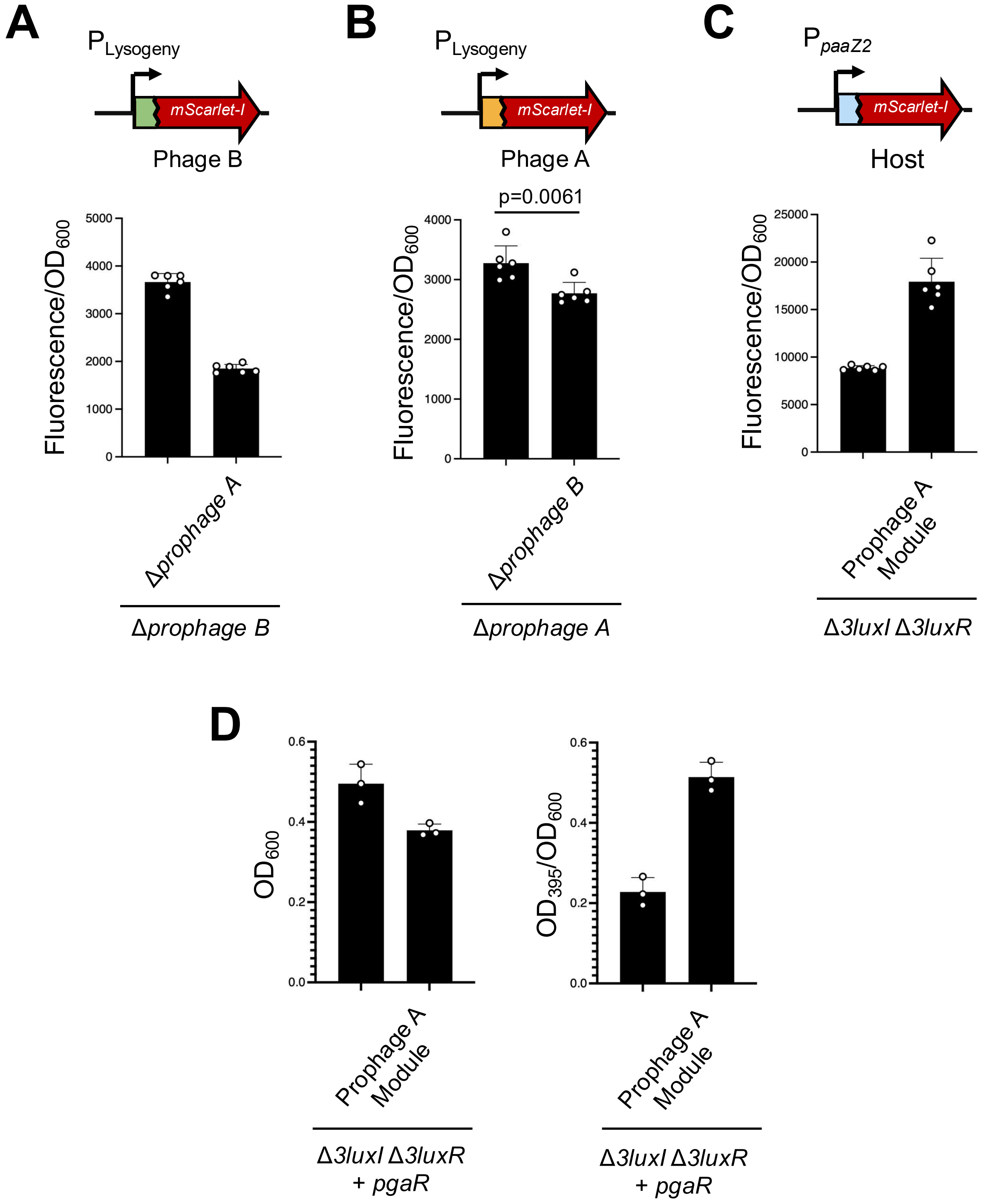

bioRxiv preprint doi: https://doi.org/10.64898/2026.07.02.736140; this version posted July 3, 2026. The copyright holder for this preprint (which was not certified by peer review) is the author/funder, who has granted bioRxiv a license to display the preprint in perpetuity. It is made available under a CC-BY 4.0 International license.

Figure S4

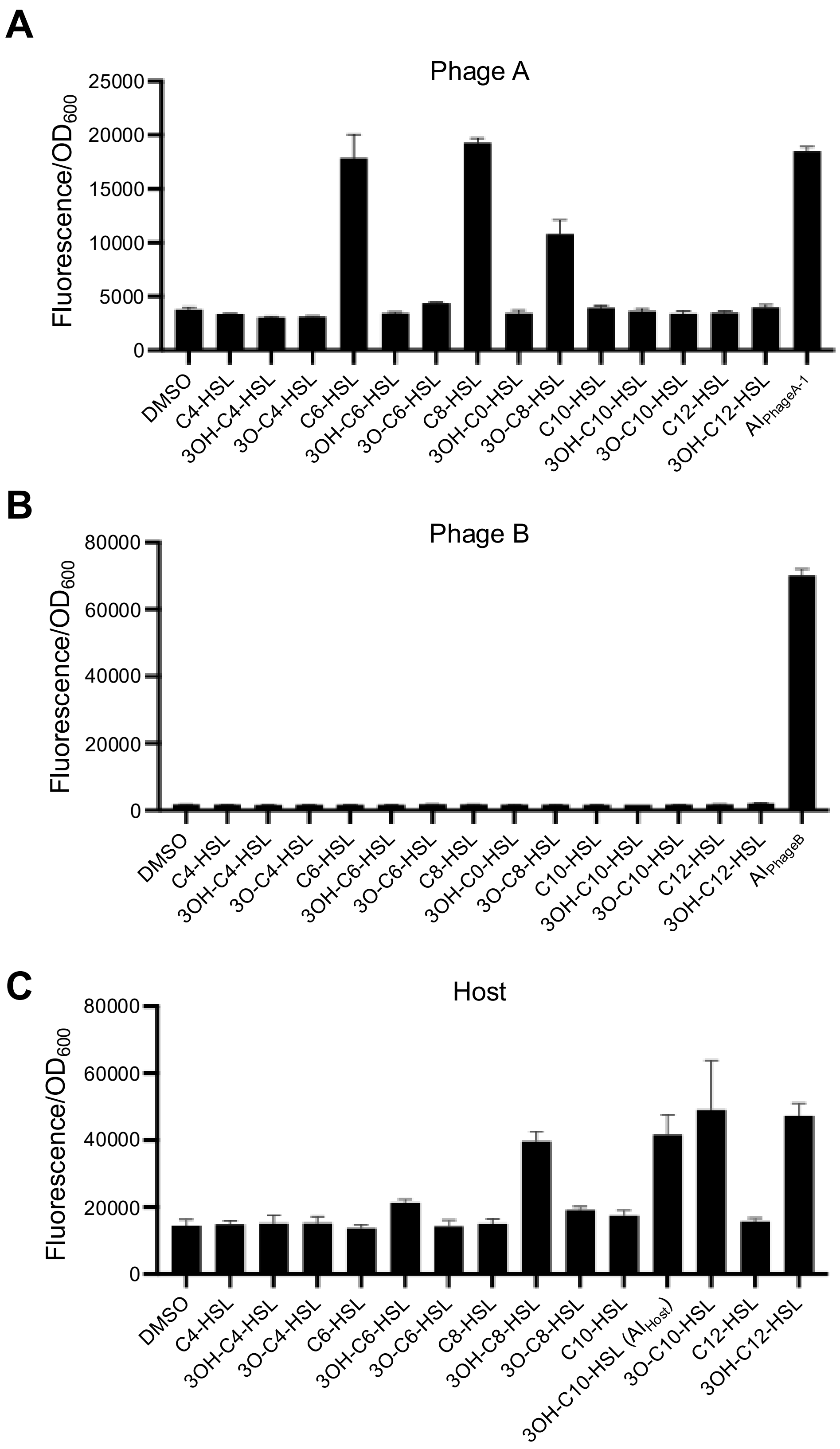

bioRxiv preprint doi: https://doi.org/10.64898/2026.07.02.736140; this version posted July 3, 2026. The copyright holder for this preprint (which was not certified by peer review) is the author/funder, who has granted bioRxiv a license to display the preprint in perpetuity. It is made available under a CC-BY 4.0 International license.

Figure S5

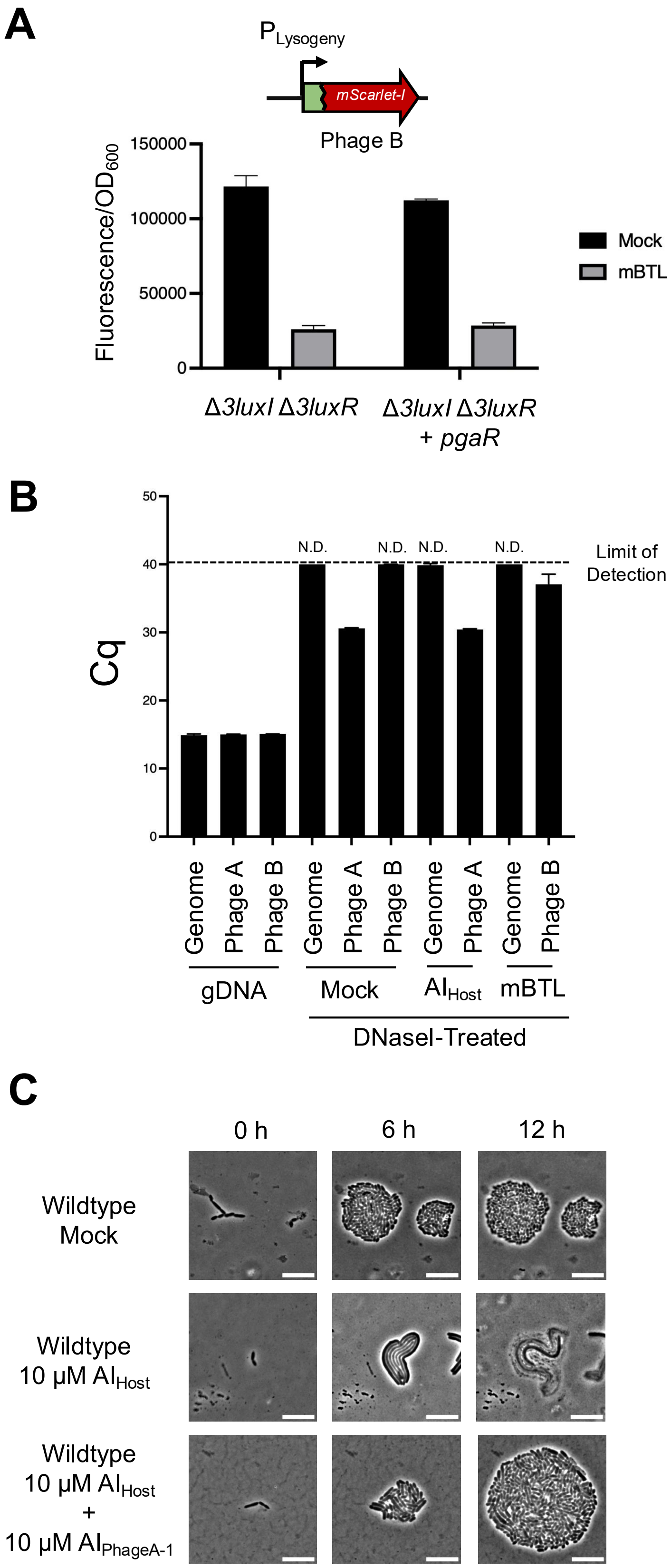

bioRxiv preprint doi: https://doi.org/10.64898/2026.07.02.736140; this version posted July 3, 2026. The copyright holder for this preprint (which was not certified by peer review) is the author/funder, who has granted bioRxiv a license to display the preprint in perpetuity. It is made available under a CC-BY 4.0 International license.

Figure S6

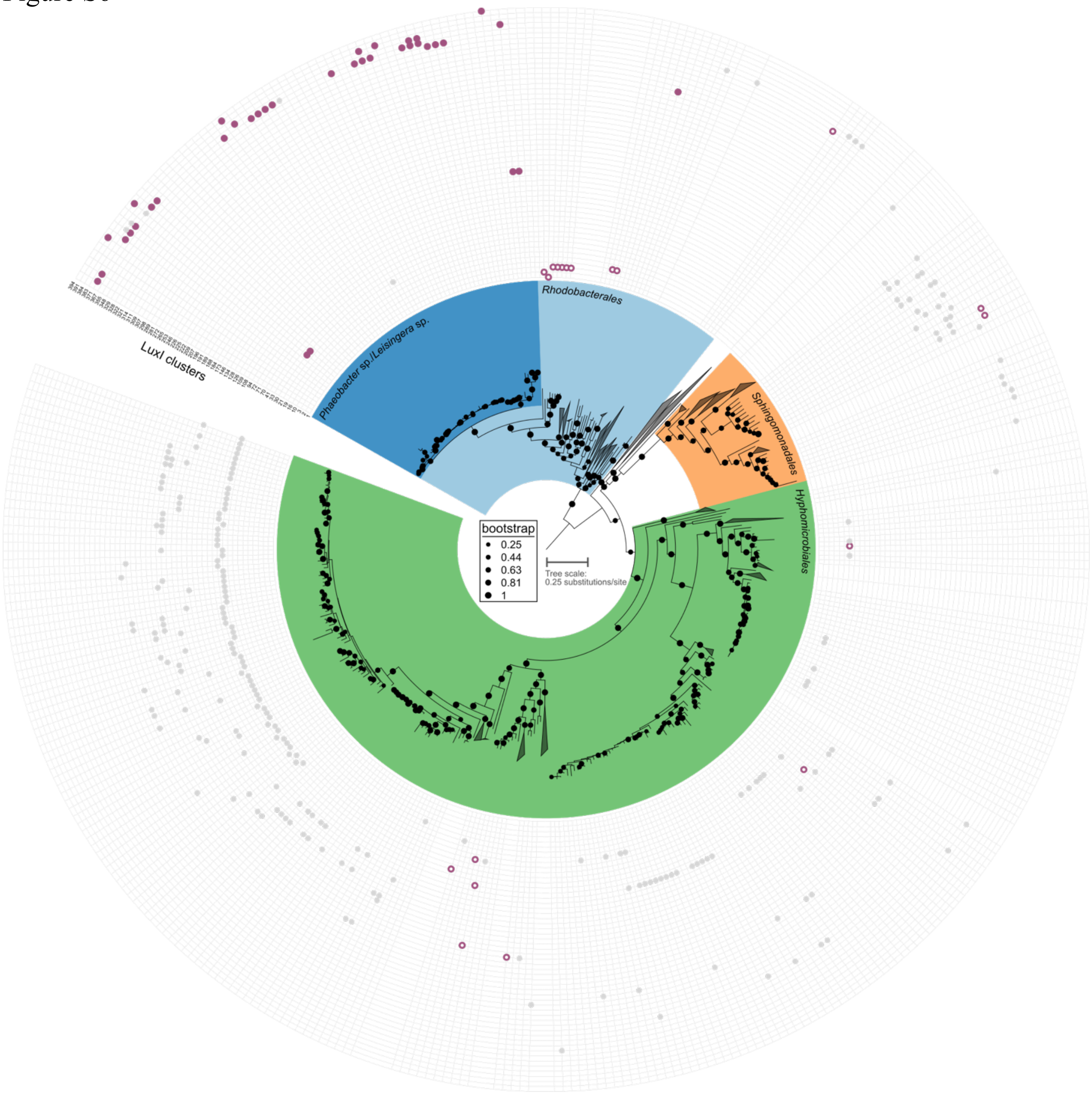

bioRxiv preprint doi: https://doi.org/10.64898/2026.07.02.736140; this version posted July 3, 2026. The copyright holder for this preprint (which was not certified by peer review) is the author/funder, who has granted bioRxiv a license to display the preprint in perpetuity. It is made available under a CC-BY 4.0 International license.

Figure S7

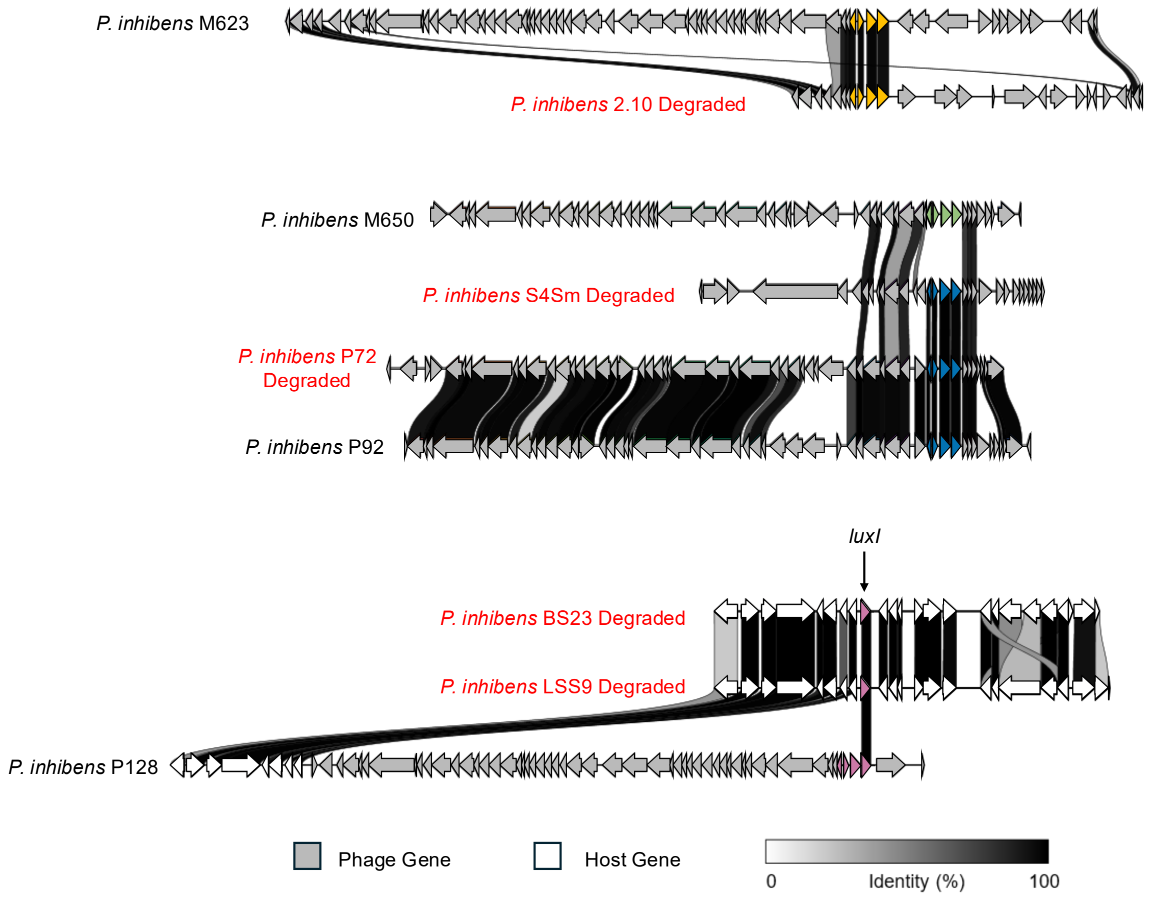

bioRxiv preprint doi: https://doi.org/10.64898/2026.07.02.736140; this version posted July 3, 2026. The copyright holder for this preprint (which was not certified by peer review) is the author/funder, who has granted bioRxiv a license to display the preprint in perpetuity. It is made available under a CC-BY 4.0 International license.

Figure S8

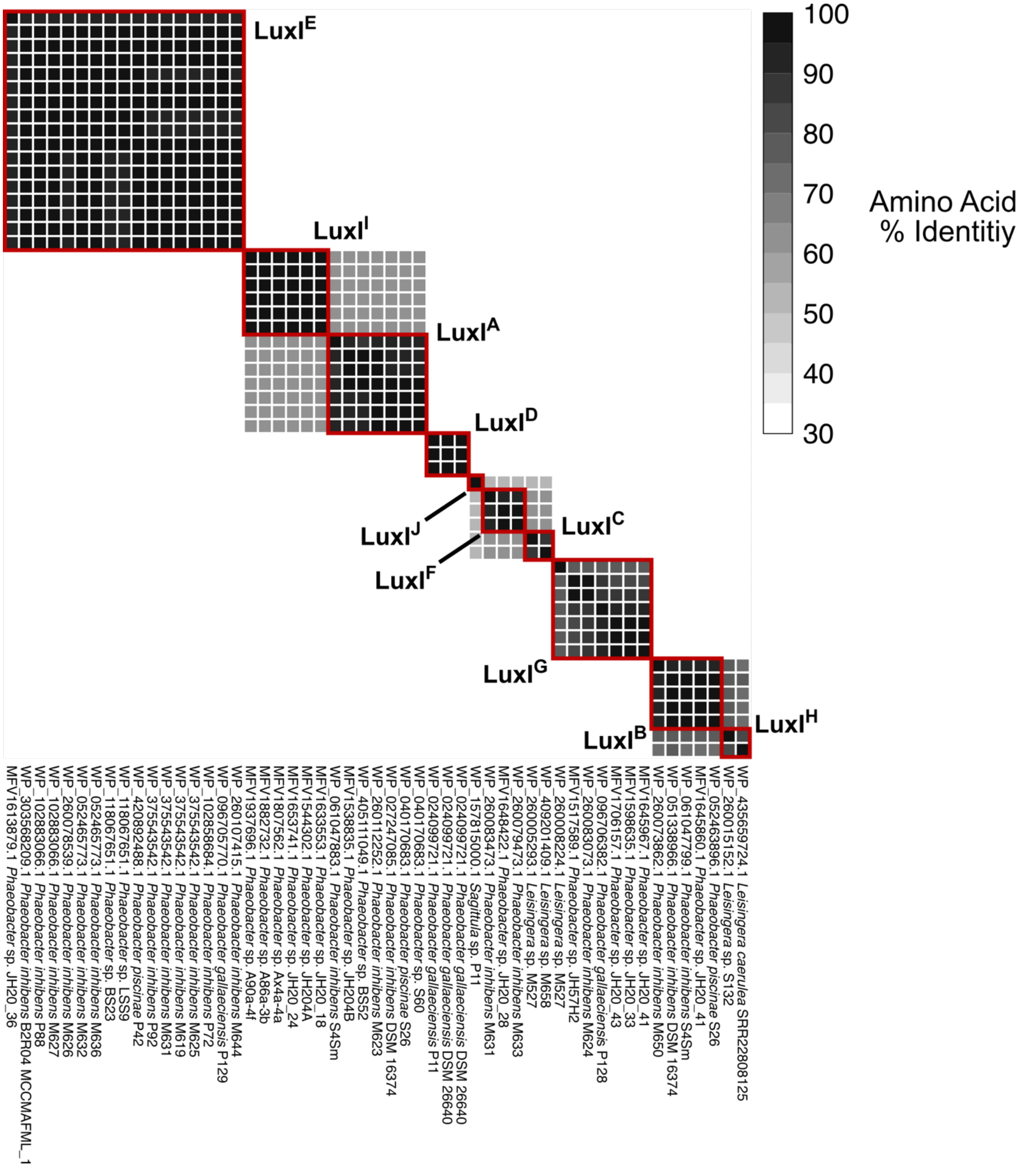

bioRxiv preprint doi: https://doi.org/10.64898/2026.07.02.736140; this version posted July 3, 2026. The copyright holder for this preprint (which was not certified by peer review) is the author/funder, who has granted bioRxiv a license to display the preprint in perpetuity. It is made available under a CC-BY 4.0 International license.

Figure S9

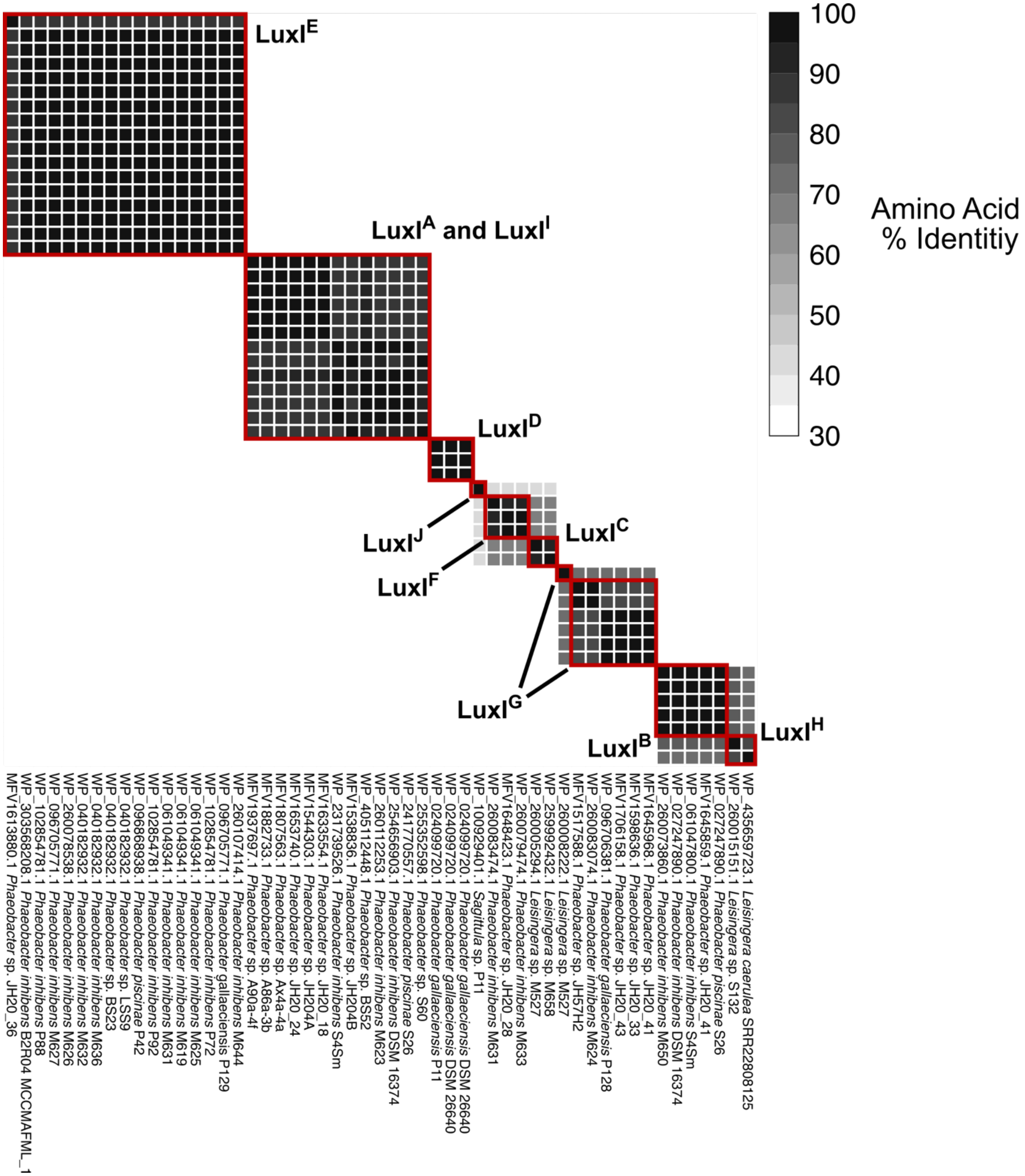

bioRxiv preprint doi: https://doi.org/10.64898/2026.07.02.736140; this version posted July 3, 2026. The copyright holder for this preprint (which was not certified by peer review) is the author/funder, who has granted bioRxiv a license to display the preprint in perpetuity. It is made available under a CC-BY 4.0 International license.

Figure S10

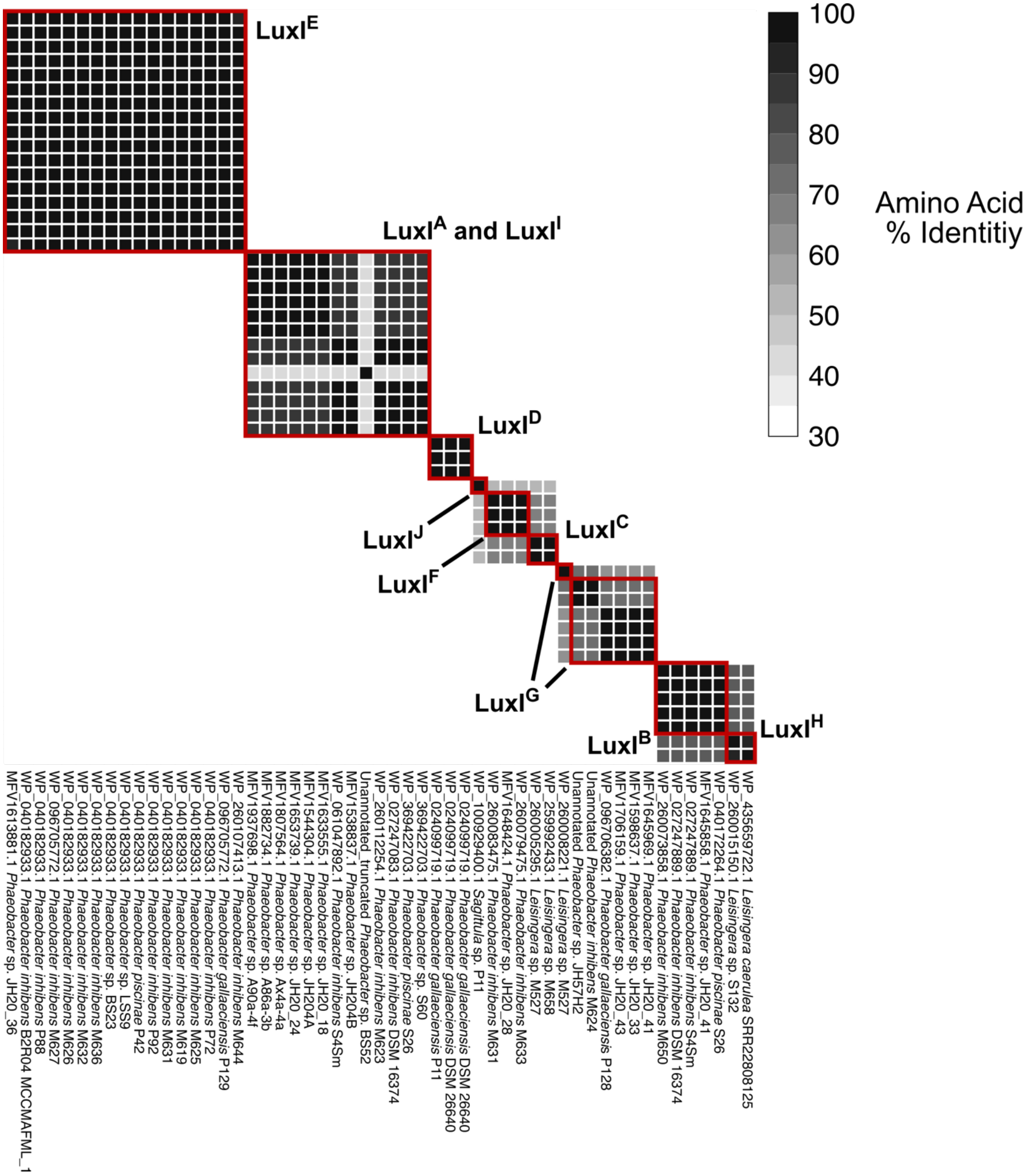

bioRxiv preprint doi: https://doi.org/10.64898/2026.07.02.736140; this version posted July 3, 2026. The copyright holder for this preprint (which was not certified by peer review) is the author/funder, who has granted bioRxiv a license to display the preprint in perpetuity. It is made available under a CC-BY 4.0 International license.

Figure S11

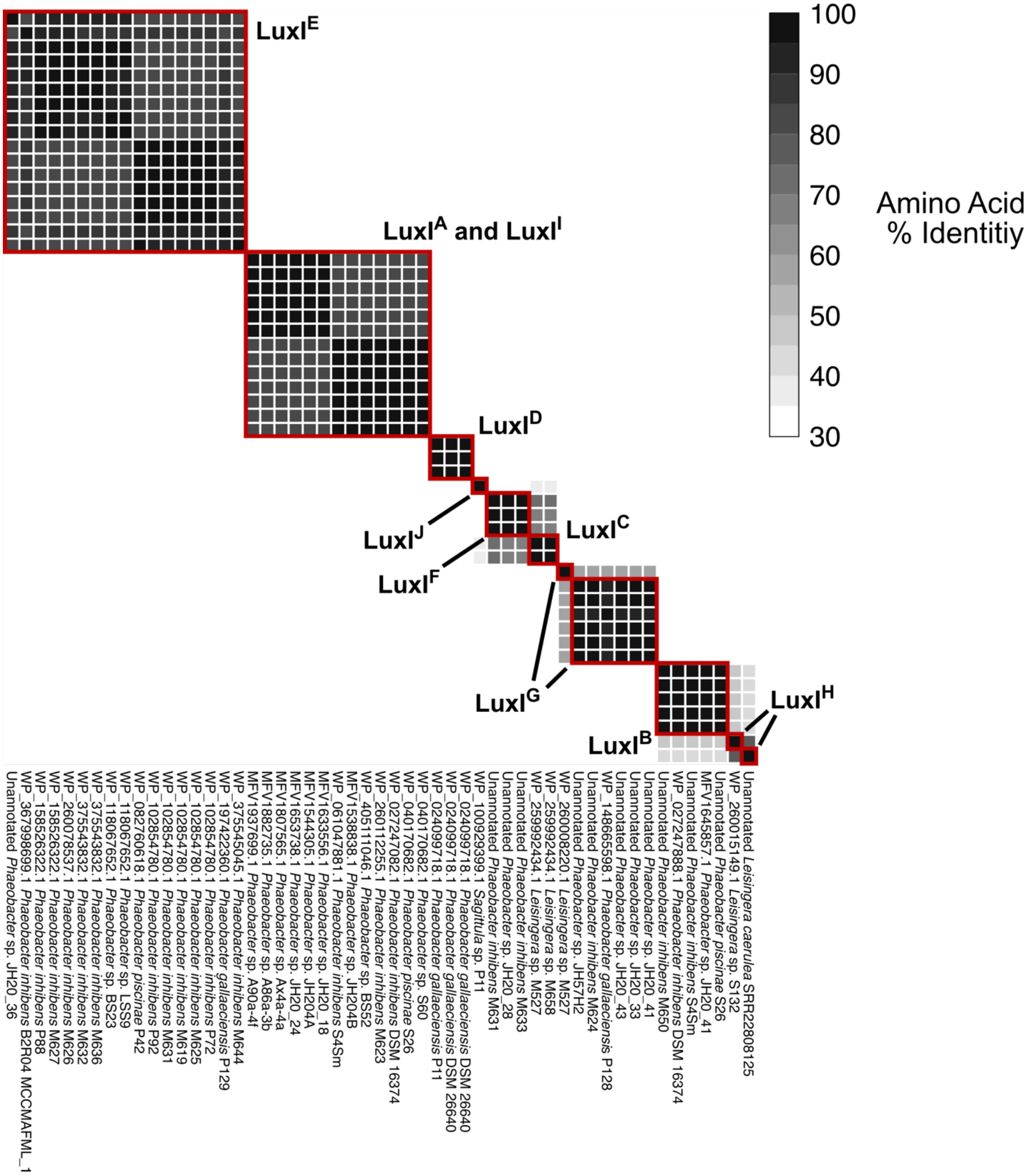

bioRxiv preprint doi: https://doi.org/10.64898/2026.07.02.736140; this version posted July 3, 2026. The copyright holder for this preprint (which was not certified by peer review) is the author/funder, who has granted bioRxiv a license to display the preprint in perpetuity. It is made available under a CC-BY 4.0 International license.

Figure S12

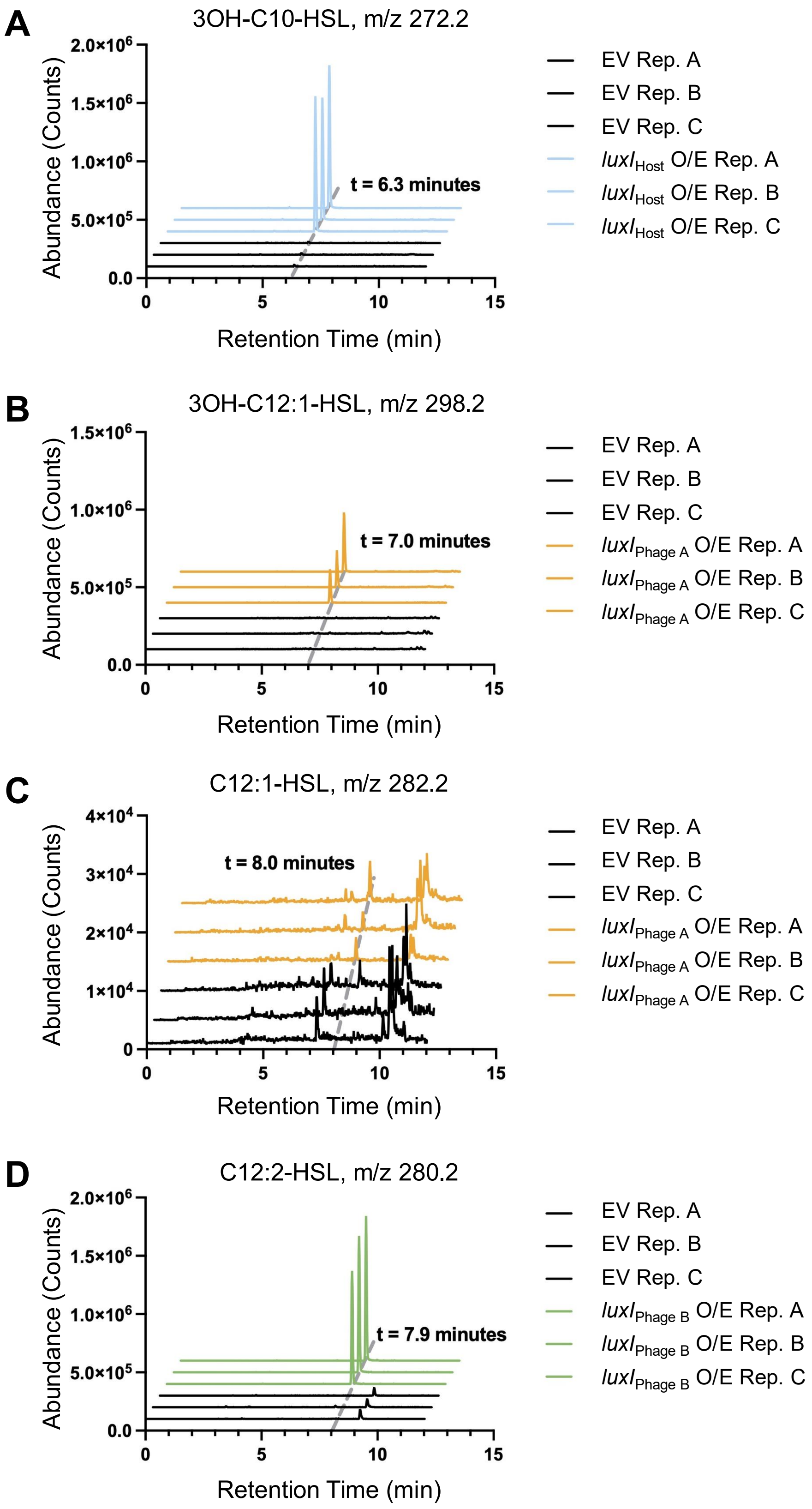

bioRxiv preprint doi: https://doi.org/10.64898/2026.07.02.736140; this version posted July 3, 2026. The copyright holder for this preprint (which was not certified by peer review) is the author/funder, who has granted bioRxiv a license to display the preprint in perpetuity. It is made available under a CC-BY 4.0 International license.

Figure S13

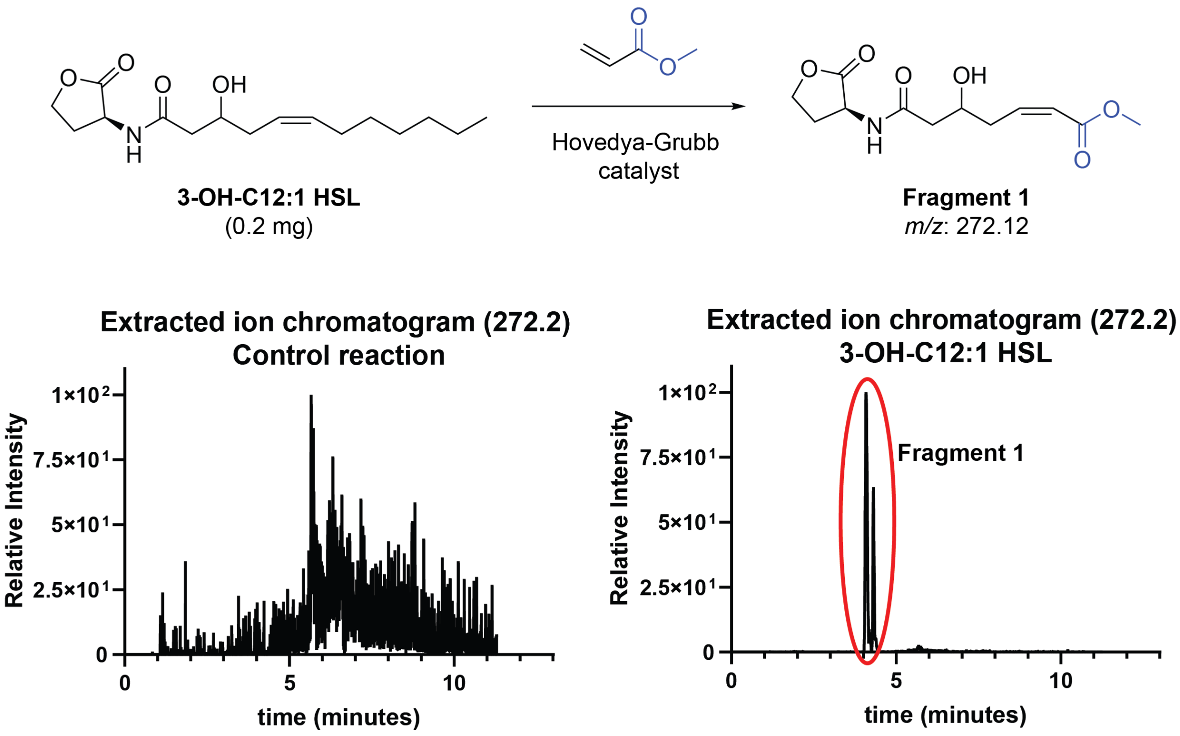

bioRxiv preprint doi: https://doi.org/10.64898/2026.07.02.736140; this version posted July 3, 2026. The copyright holder for this preprint (which was not certified by peer review) is the author/funder, who has granted bioRxiv a license to display the preprint in perpetuity. It is made available under a CC-BY 4.0 International license.

Figure S14

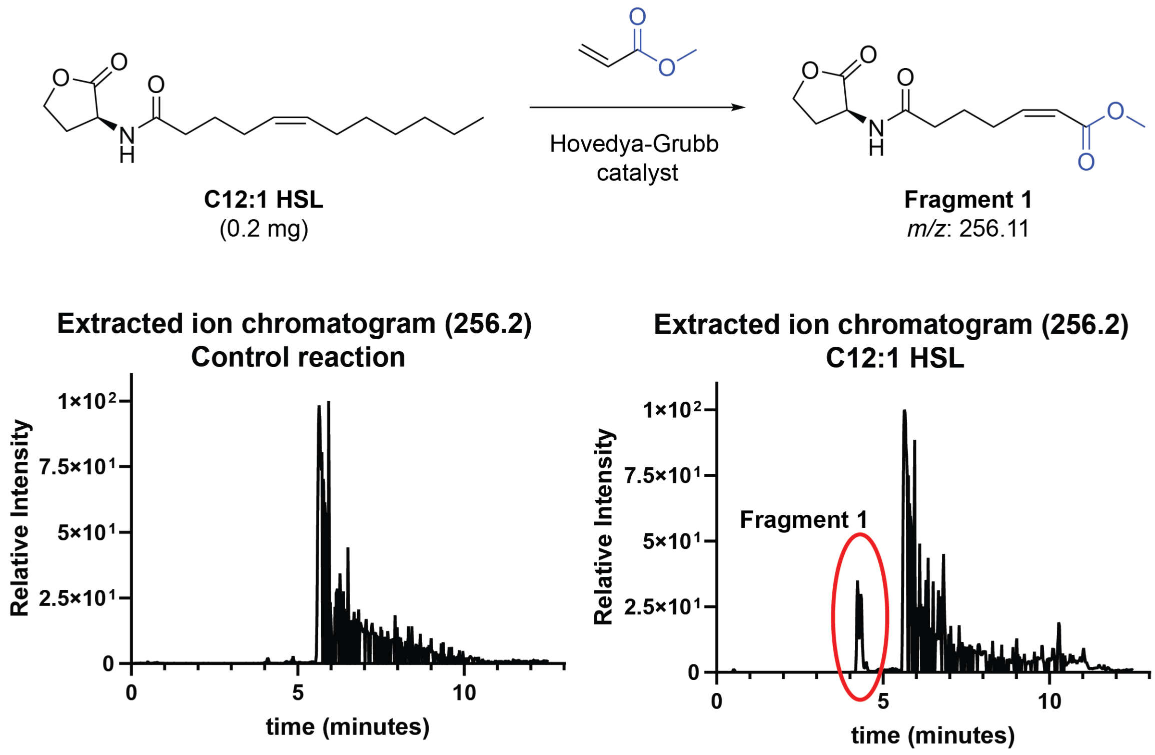

bioRxiv preprint doi: https://doi.org/10.64898/2026.07.02.736140; this version posted July 3, 2026. The copyright holder for this preprint (which was not certified by peer review) is the author/funder, who has granted bioRxiv a license to display the preprint in perpetuity. It is made available under a CC-BY 4.0 International license.

Figure S15

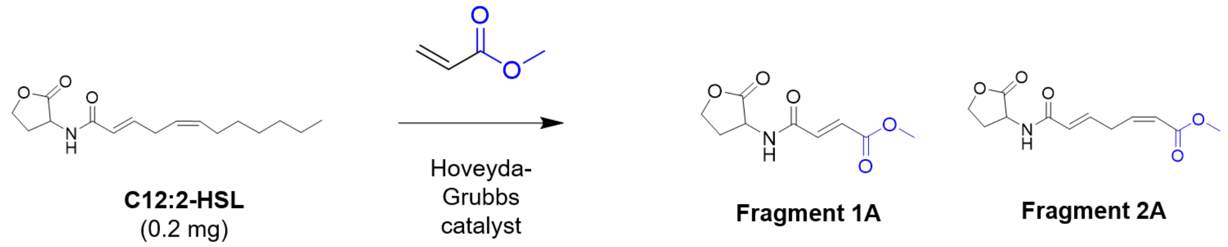

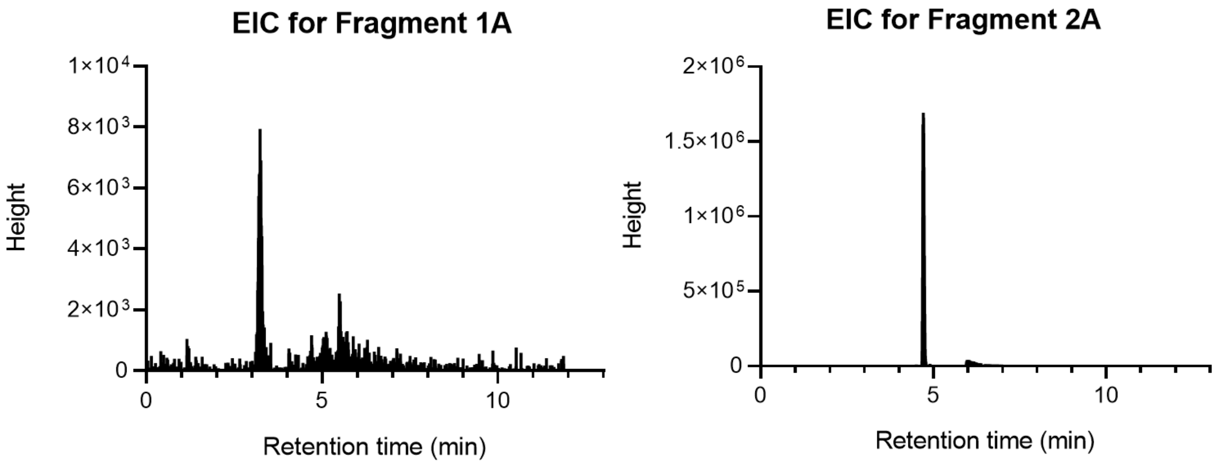

bioRxiv preprint doi: https://doi.org/10.64898/2026.07.02.736140; this version posted July 3, 2026. The copyright holder for this preprint (which was not certified by peer review) is the author/funder, who has granted bioRxiv a license to display the preprint in perpetuity. It is made available under a CC-BY 4.0 International license.

Figure S16

**Position**	**δ_C**	**δ_H**
**1**	175.6	
**2**	66.3	4.48, m<br>4.29, m
**3**	30.9	2.89, m<br>2.16, m
**4**	49.5	4.60, ddd (11.5, 8.5, 5.5)
**4-NH**		6.05, d (5.5)
**5**	166.5	
**6**	122.5	5.84, d (15.0)
**7**	144.9	6.90, dt (15.0, 6.2)
**8**	29.9	2.94, dd (7.3, 6.2)
**9**	124.2	5.36, dt (10.6, 7.3)
**10**	133.1	5.54, dt (10.6, 7.3)
**11**	27.4	2.01, dt (7.3, 7.3)
**12**	29.6	1.34, m
**13**	29.1	1.27, m
**14**	31.9	1.26, m
**15**	22.8	1.29, m
**16**	14.2	0.88, t (7.0)

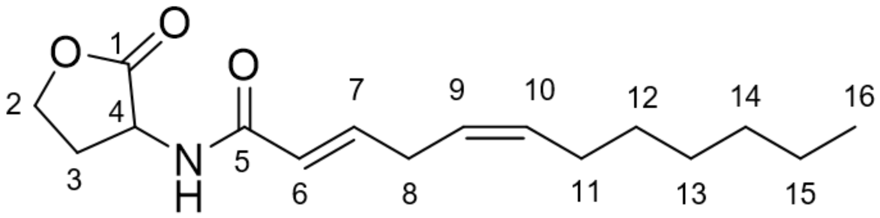

bioRxiv preprint doi: https://doi.org/10.64898/2026.07.02.736140; this version posted July 3, 2026. The copyright holder for this preprint (which was not certified by peer review) is the author/funder, who has granted bioRxiv a license to display the preprint in perpetuity. It is made available under a CC-BY 4.0 International license.

Figure S17

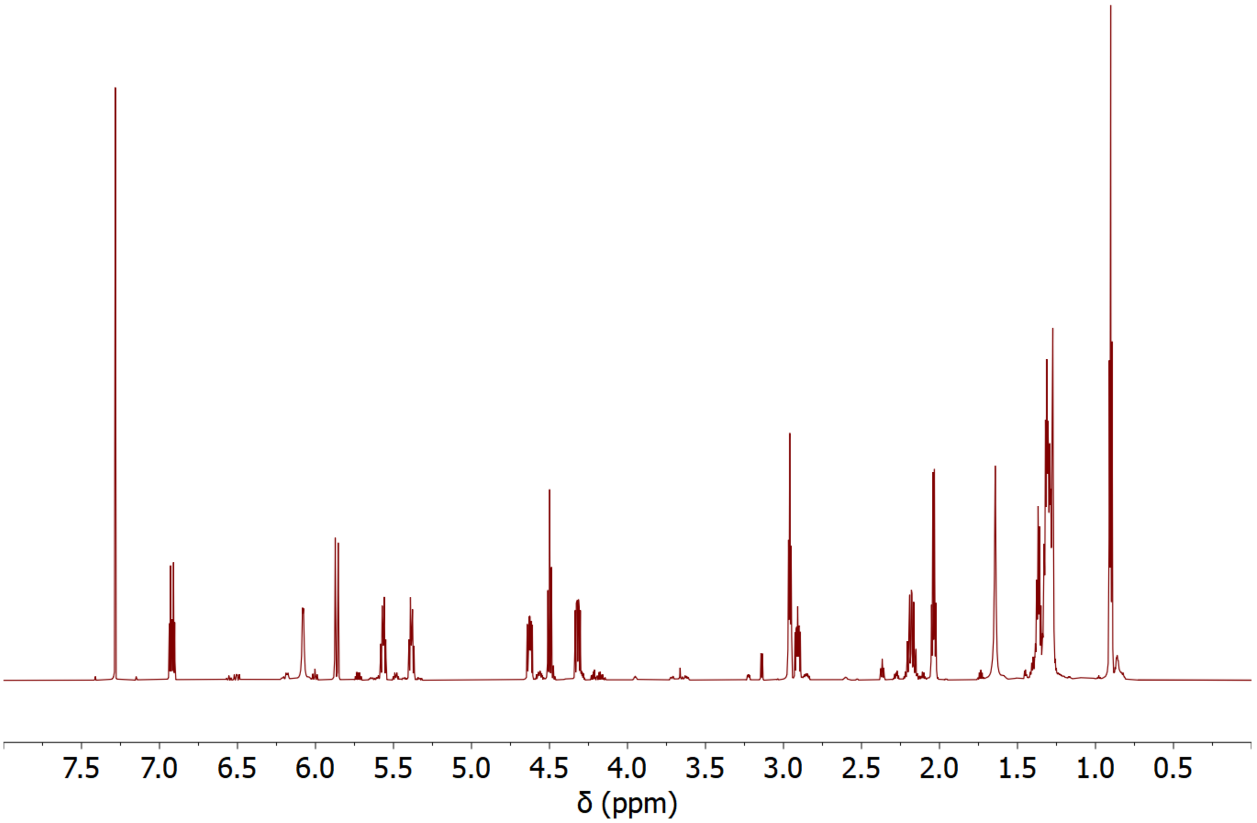

bioRxiv preprint doi: https://doi.org/10.64898/2026.07.02.736140; this version posted July 3, 2026. The copyright holder for this preprint (which was not certified by peer review) is the author/funder, who has granted bioRxiv a license to display the preprint in perpetuity. It is made available under a CC-BY 4.0 International license.

Figure S18

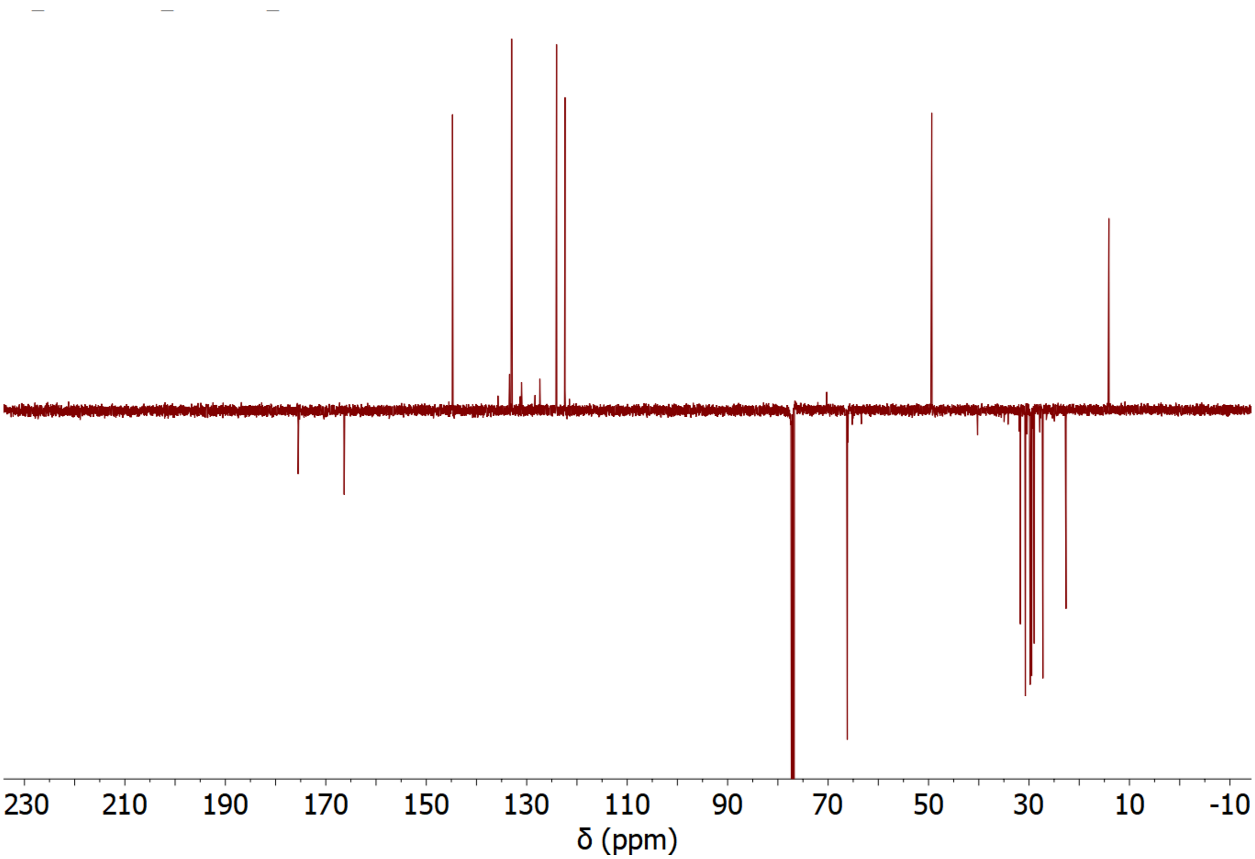

bioRxiv preprint doi: https://doi.org/10.64898/2026.07.02.736140; this version posted July 3, 2026. The copyright holder for this preprint (which was not certified by peer review) is the author/funder, who has granted bioRxiv a license to display the preprint in perpetuity. It is made available under a CC-BY 4.0 International license.

Figure S19

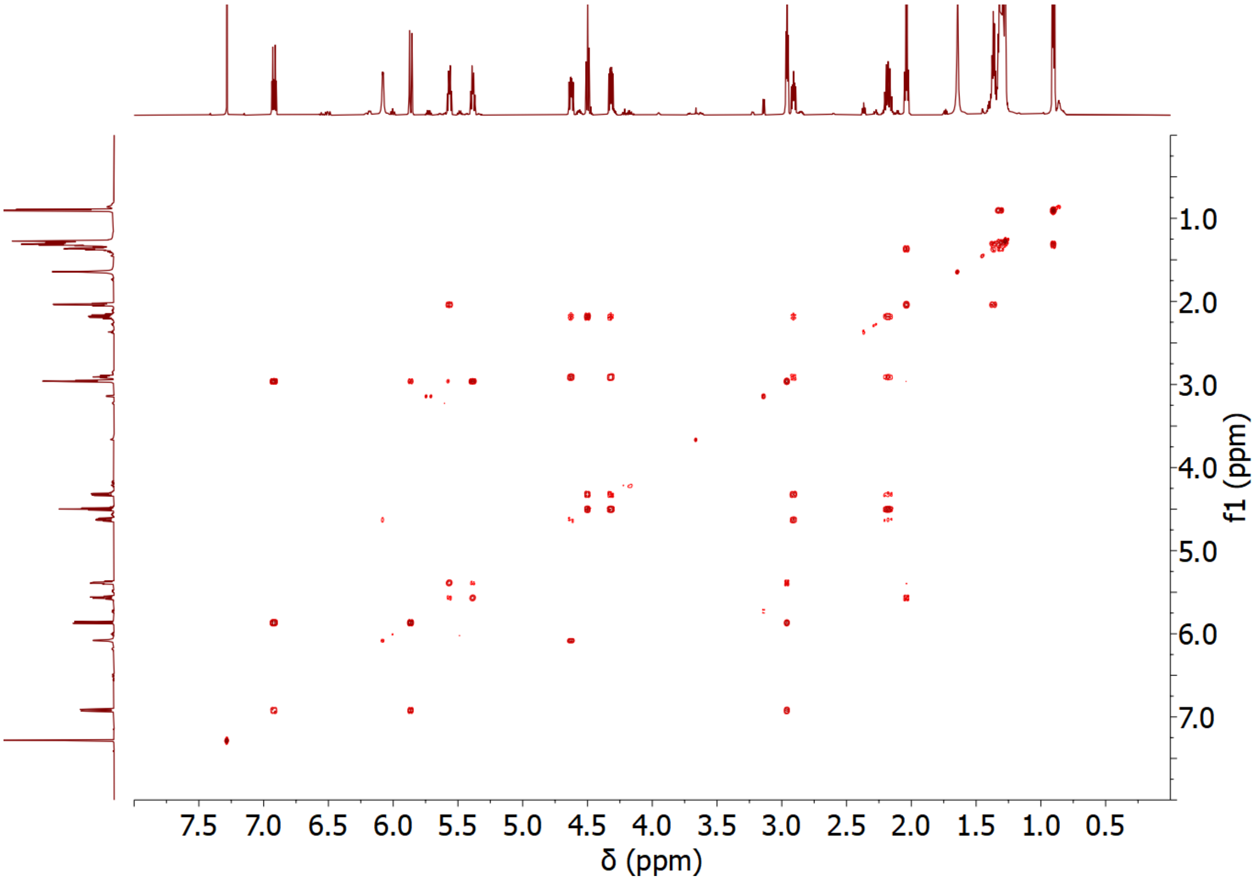

bioRxiv preprint doi: https://doi.org/10.64898/2026.07.02.736140; this version posted July 3, 2026. The copyright holder for this preprint (which was not certified by peer review) is the author/funder, who has granted bioRxiv a license to display the preprint in perpetuity. It is made available under a CC-BY 4.0 International license.

Figure S20

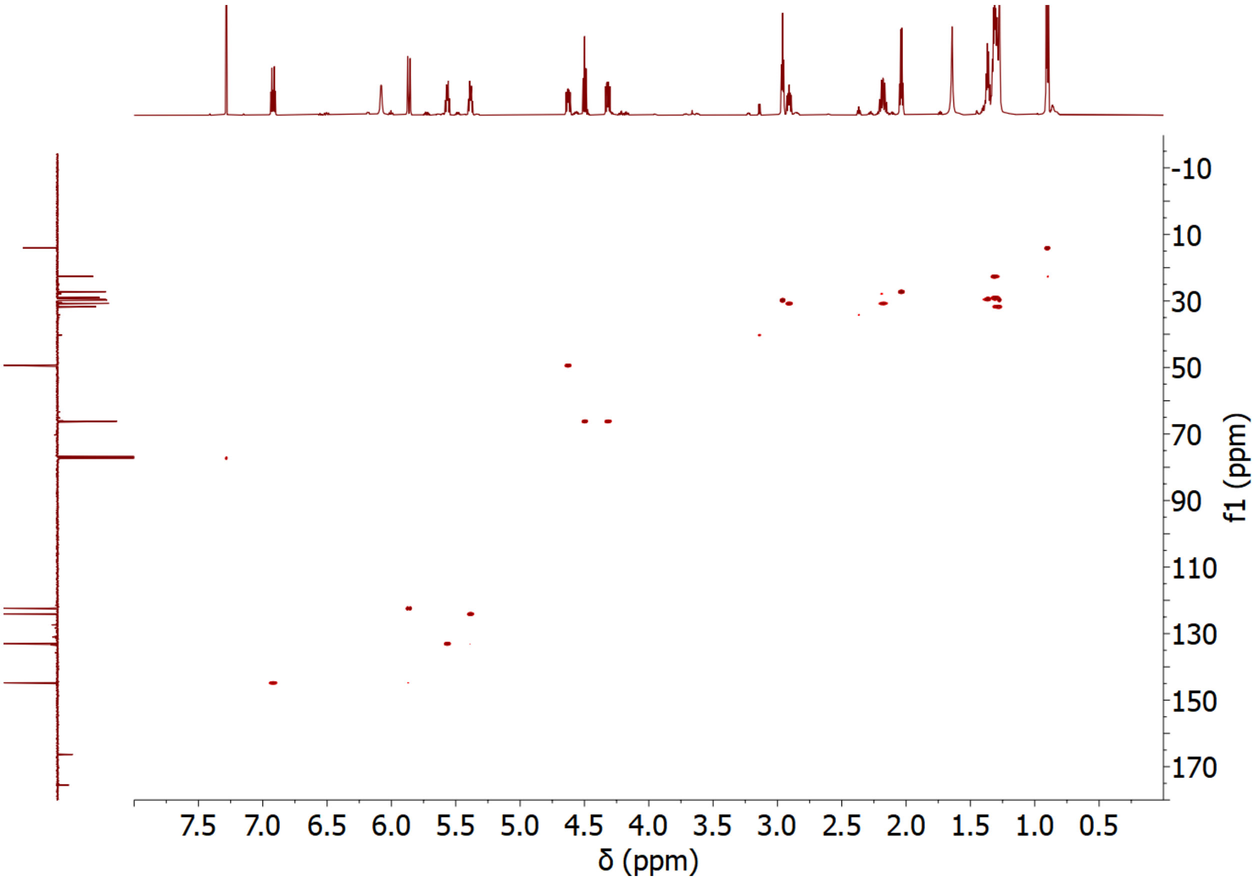

bioRxiv preprint doi: https://doi.org/10.64898/2026.07.02.736140; this version posted July 3, 2026. The copyright holder for this preprint (which was not certified by peer review) is the author/funder, who has granted bioRxiv a license to display the preprint in perpetuity. It is made available under a CC-BY 4.0 International license.

Figure S21

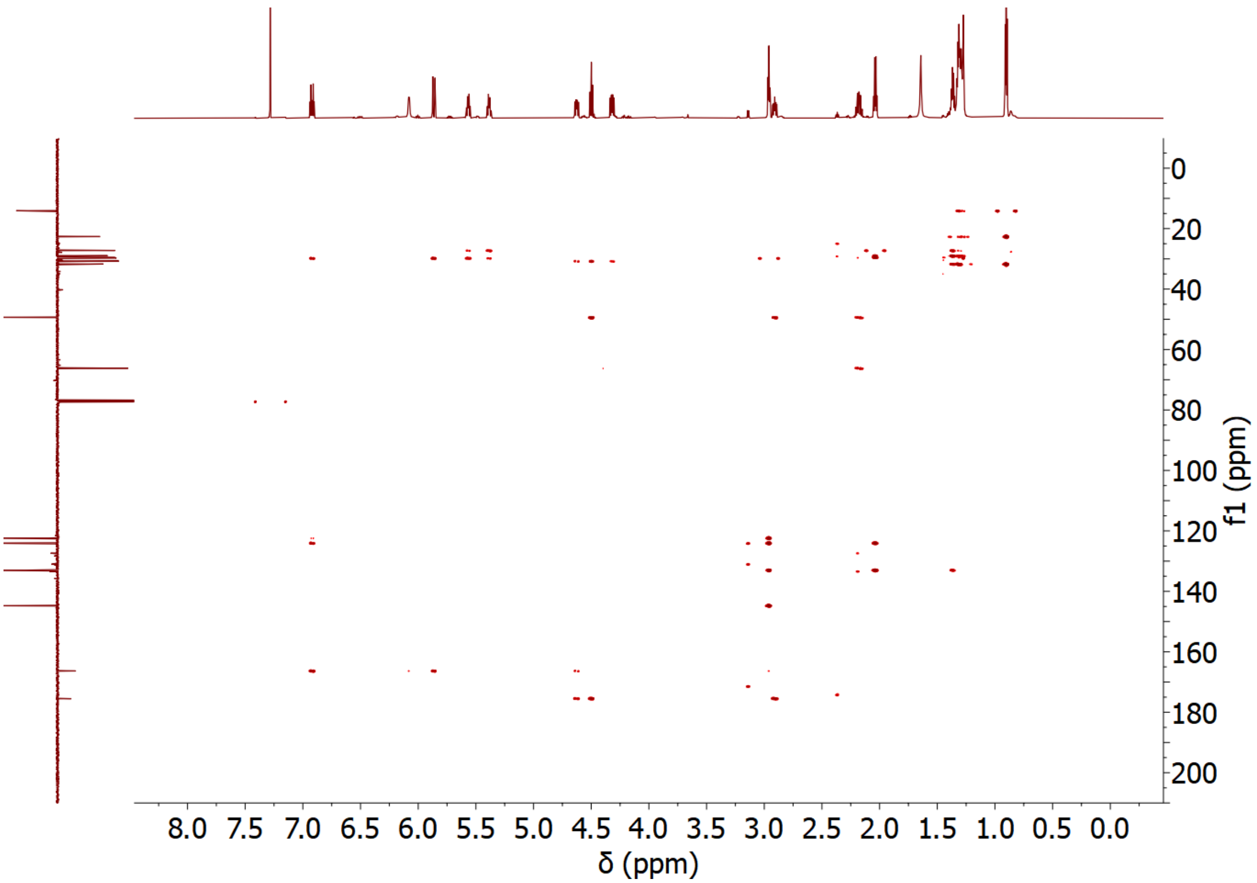

bioRxiv preprint doi: https://doi.org/10.64898/2026.07.02.736140; this version posted July 3, 2026. The copyright holder for this preprint (which was not certified by peer review) is the author/funder, who has granted bioRxiv a license to display the preprint in perpetuity. It is made available under a CC-BY 4.0 International license.

Figure S22

Position	δ_C	δ_H
1	175.2	
2	65.9	4.31, d (7.66)<br>4.50, ddd (5.90, 9.32, 11.26)
3	30.5	2.20, m<br>2.87, m
4	52.6	3.82, dddd (1.22, 5.95, 8.61, 12.59)
5	172.8	
6	30.5	2.10, , qd (1.60, 7.33, 7.42, 7.42)<br>2.50, dd (6.30, 12.71)
7	49.2	4.58, td (1.23, 9.07, 8.99)
7-OH		6.77, m
8	29.4	2.07, qd (1.60, 7.33, 7.42, 7.42)
9	134.3	5.62, dtt (1.66, 1.66, 7.46, 7.46, 10.86)
10	123.7	5.42, ddd (5.90, 9.32, 11.26)
11	27.5	2.32, m
12	22.7	1.31, m
13	29.4	1.32, m
14	31.8	1.30, m
15	17.4	1.32, m
16	14.2	0.91, m

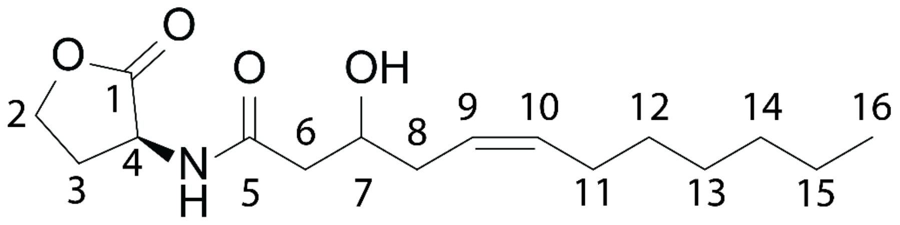

Chemical Formula: $C_{16}H_{27}NO_4$
Molecular Weight: 297.40

bioRxiv preprint doi: https://doi.org/10.64898/2026.07.02.736140; this version posted July 3, 2026. The copyright holder for this preprint (which was not certified by peer review) is the author/funder, who has granted bioRxiv a license to display the preprint in perpetuity. It is made available under a CC-BY 4.0 International license.

Figure S23

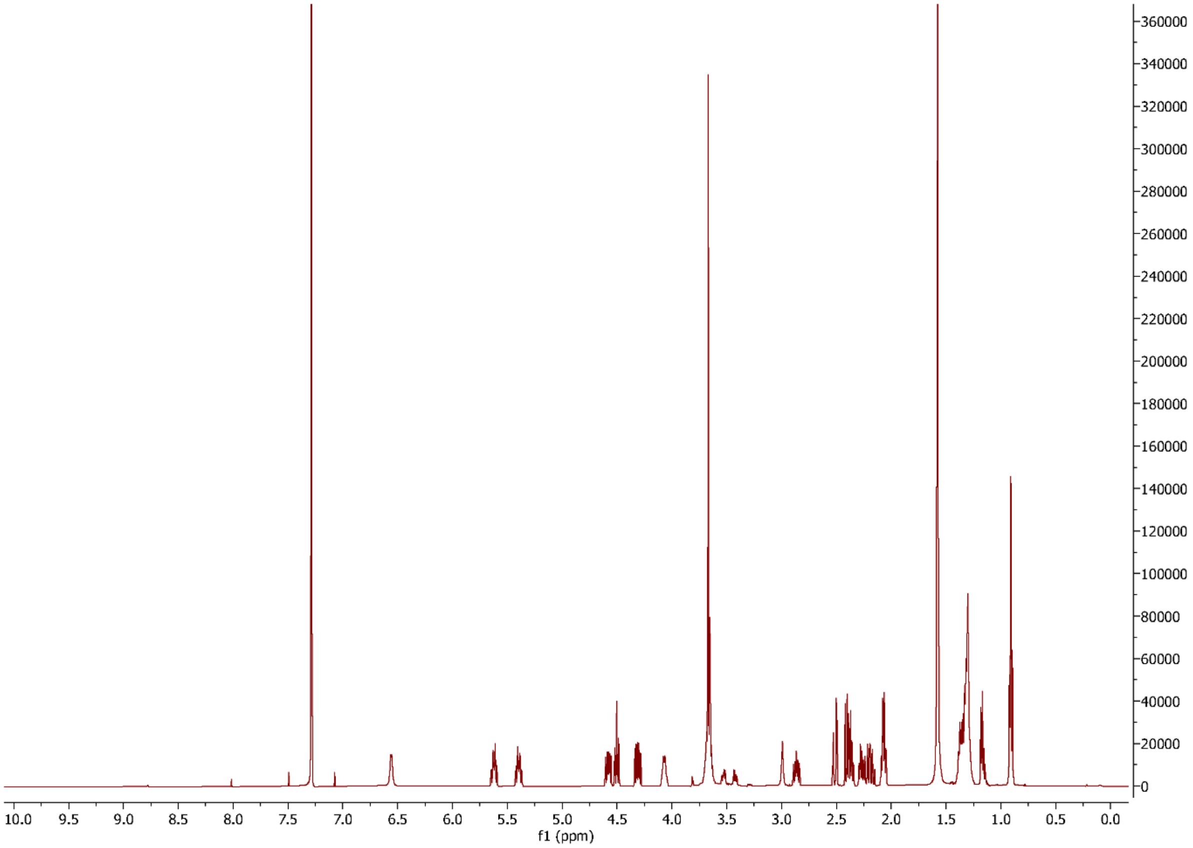

bioRxiv preprint doi: https://doi.org/10.64898/2026.07.02.736140; this version posted July 3, 2026. The copyright holder for this preprint (which was not certified by peer review) is the author/funder, who has granted bioRxiv a license to display the preprint in perpetuity. It is made available under a CC-BY 4.0 International license.

Figure S24

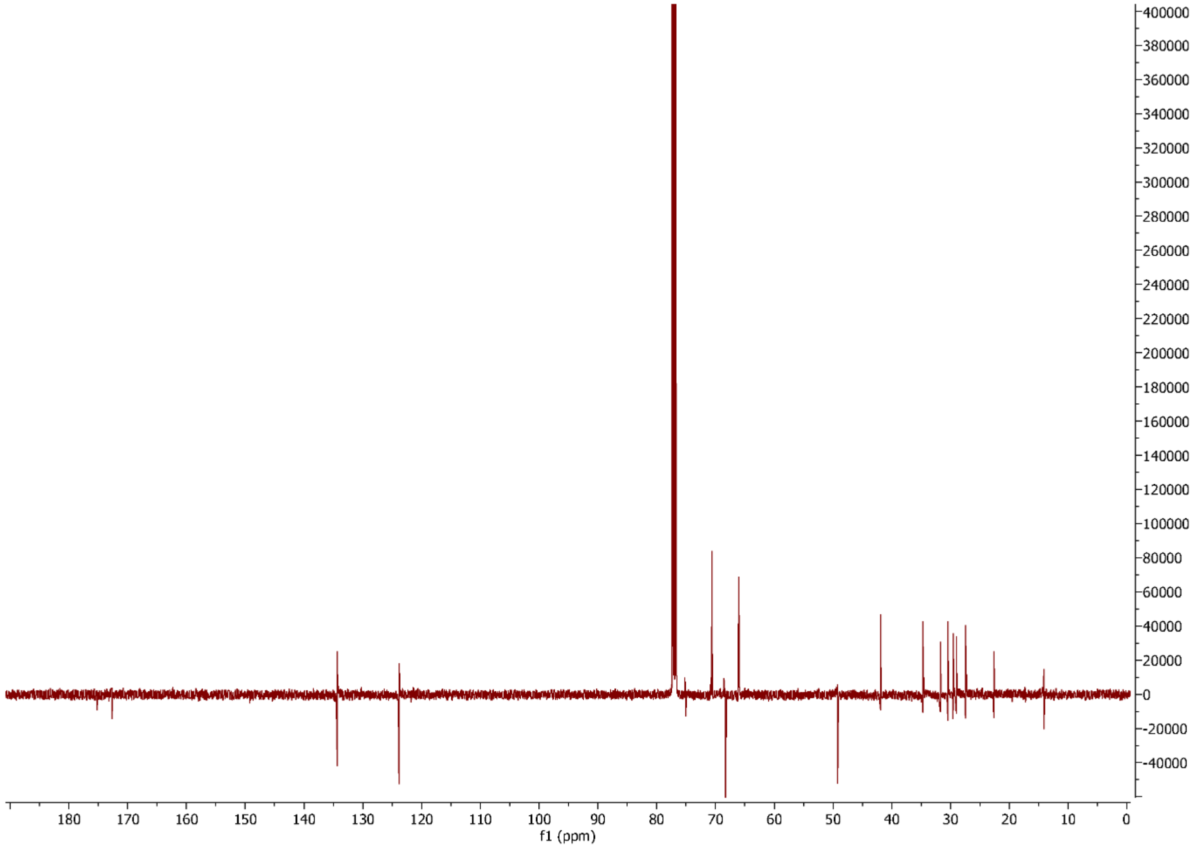

bioRxiv preprint doi: https://doi.org/10.64898/2026.07.02.736140; this version posted July 3, 2026. The copyright holder for this preprint (which was not certified by peer review) is the author/funder, who has granted bioRxiv a license to display the preprint in perpetuity. It is made available under a CC-BY 4.0 International license.

Figure S25

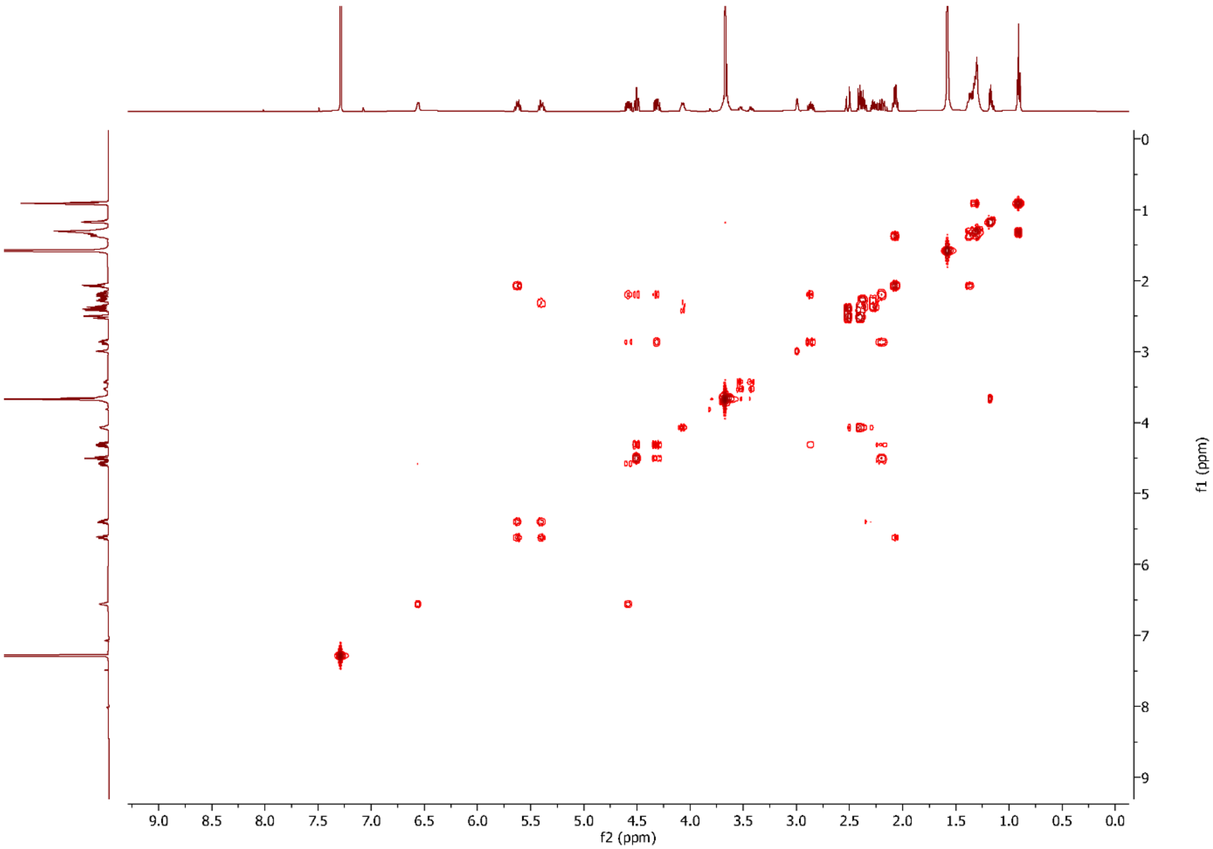

bioRxiv preprint doi: https://doi.org/10.64898/2026.07.02.736140; this version posted July 3, 2026. The copyright holder for this preprint (which was not certified by peer review) is the author/funder, who has granted bioRxiv a license to display the preprint in perpetuity. It is made available under a CC-BY 4.0 International license.

Figure S26

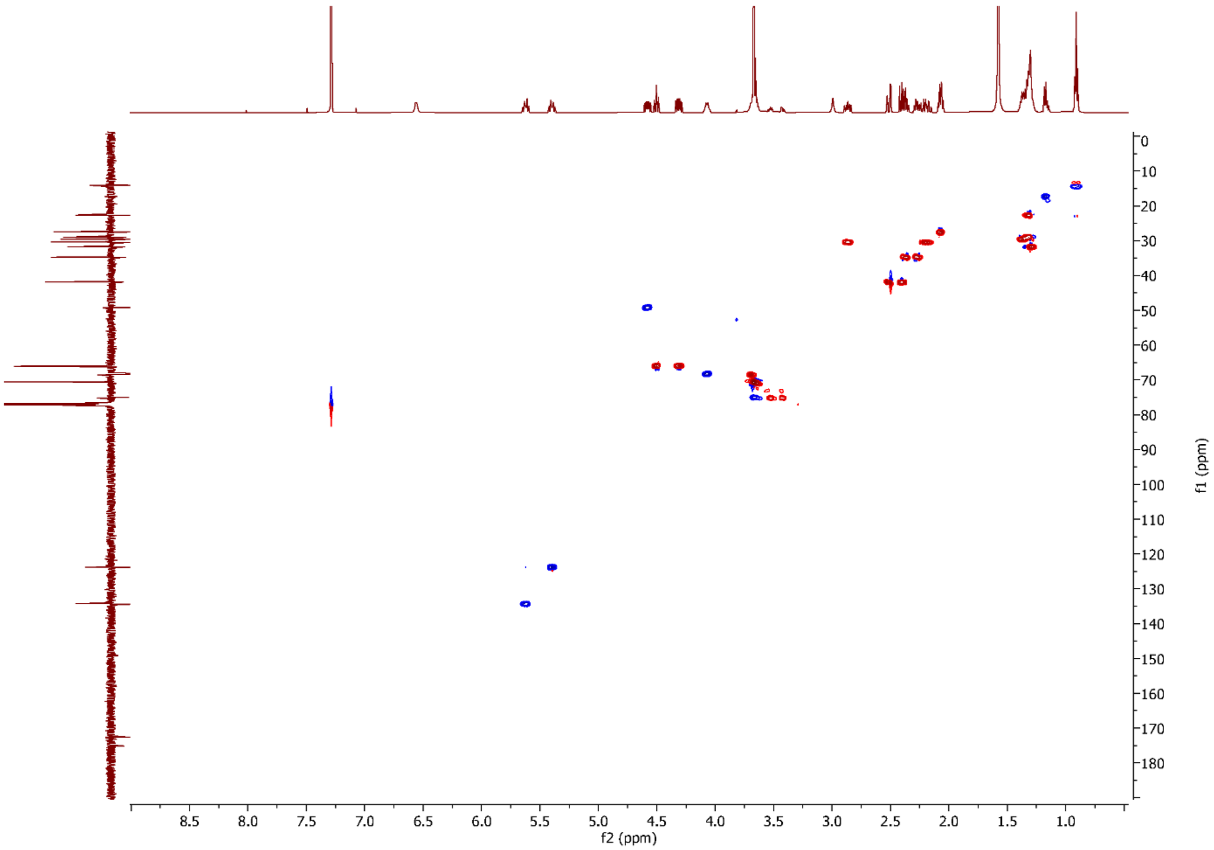

bioRxiv preprint doi: https://doi.org/10.64898/2026.07.02.736140; this version posted July 3, 2026. The copyright holder for this preprint (which was not certified by peer review) is the author/funder, who has granted bioRxiv a license to display the preprint in perpetuity. It is made available under a CC-BY 4.0 International license.

Figure S27

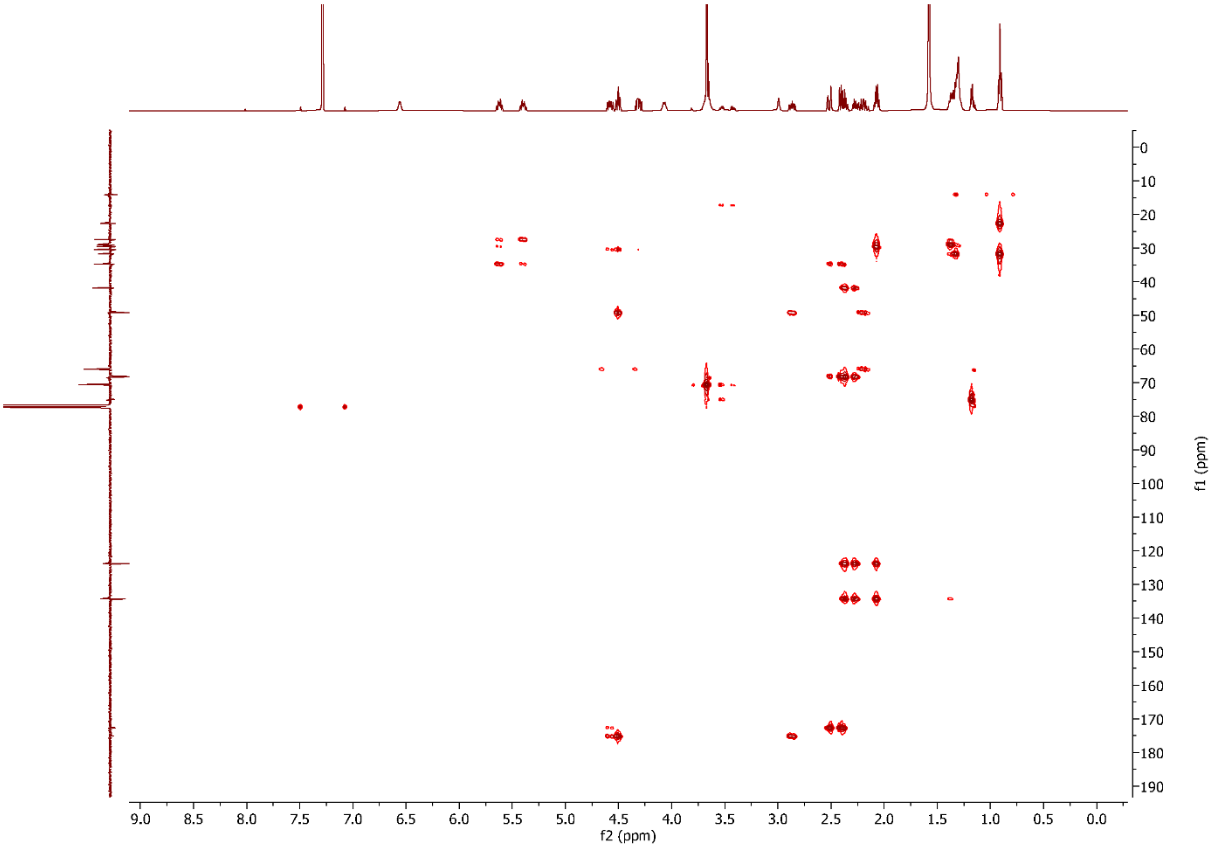

bioRxiv preprint doi: https://doi.org/10.64898/2026.07.02.736140; this version posted July 3, 2026. The copyright holder for this preprint (which was not certified by peer review) is the author/funder, who has granted bioRxiv a license to display the preprint in perpetuity. It is made available under a CC-BY 4.0 International license.

Figure S28

Position	δ_C	δ_H
1	175.5	
2	66.2	4.31, dt (7.76, 7.76, 15.97)<br>4.50, m
3	30.9	2.92, m<br>2.14, m
4	49.4	4.55, m
5	173.6	
6	35.6	2.28, m
7	25.3	1.75, m
8	26.4	2.13, p (9.75, 9.75, 8.33, 8.33)
9	128.2	5.35, q (8.67, 8.67, 9.53)
10	131.3	5.45, q (8.57, 8.57, 9.53)
11	27.2	2.04, q (7.49, 7.49, 7.71)
12	31.8	1.33, dd (10.32, 18.02)
13	29.62	1.3, dd (10.32, 18.02)
14	28.98	1.3, dd (10.32, 18.02)
15	22.73	1.31, dd (10.32, 18.02)
16	14.2	0.91, m

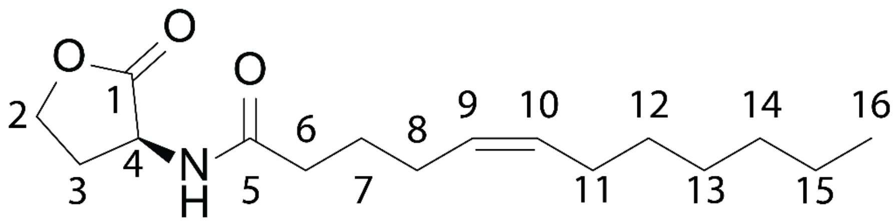

Chemical Formula: $C_{16}H_{27}NO_3$
Molecular Weight: 281.40

bioRxiv preprint doi: https://doi.org/10.64898/2026.07.02.736140; this version posted July 3, 2026. The copyright holder for this preprint (which was not certified by peer review) is the author/funder, who has granted bioRxiv a license to display the preprint in perpetuity. It is made available under a CC-BY 4.0 International license.

Figure S29

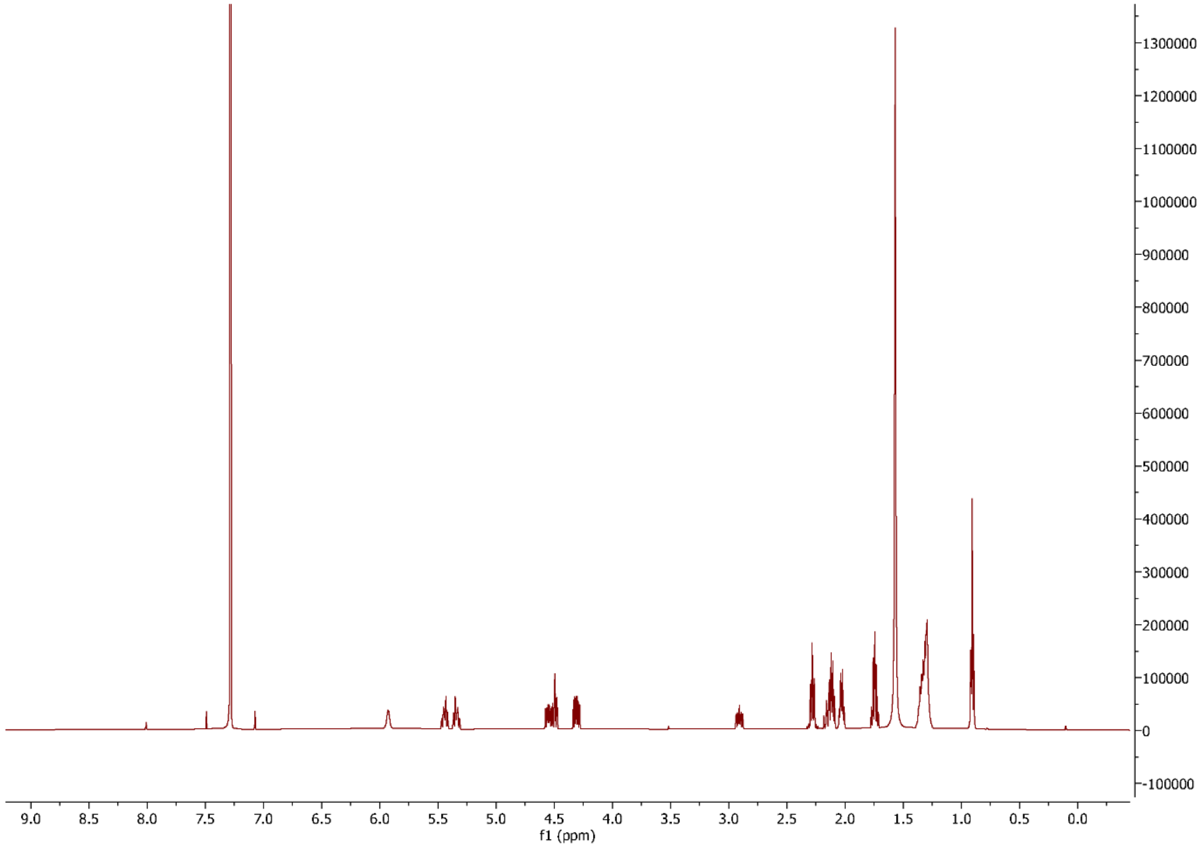

bioRxiv preprint doi: https://doi.org/10.64898/2026.07.02.736140; this version posted July 3, 2026. The copyright holder for this preprint (which was not certified by peer review) is the author/funder, who has granted bioRxiv a license to display the preprint in perpetuity. It is made available under a CC-BY 4.0 International license.

Figure S30

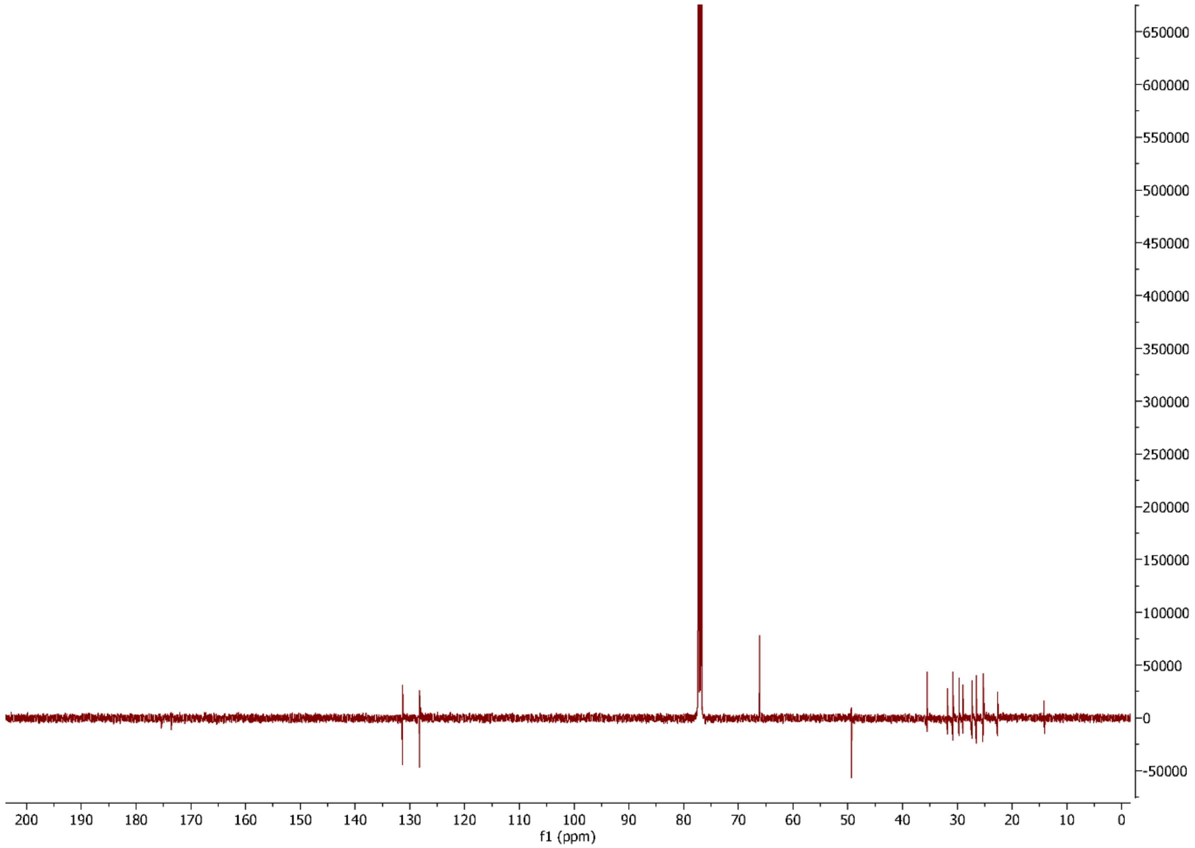

bioRxiv preprint doi: https://doi.org/10.64898/2026.07.02.736140; this version posted July 3, 2026. The copyright holder for this preprint (which was not certified by peer review) is the author/funder, who has granted bioRxiv a license to display the preprint in perpetuity. It is made available under a CC-BY 4.0 International license.

Figure S31

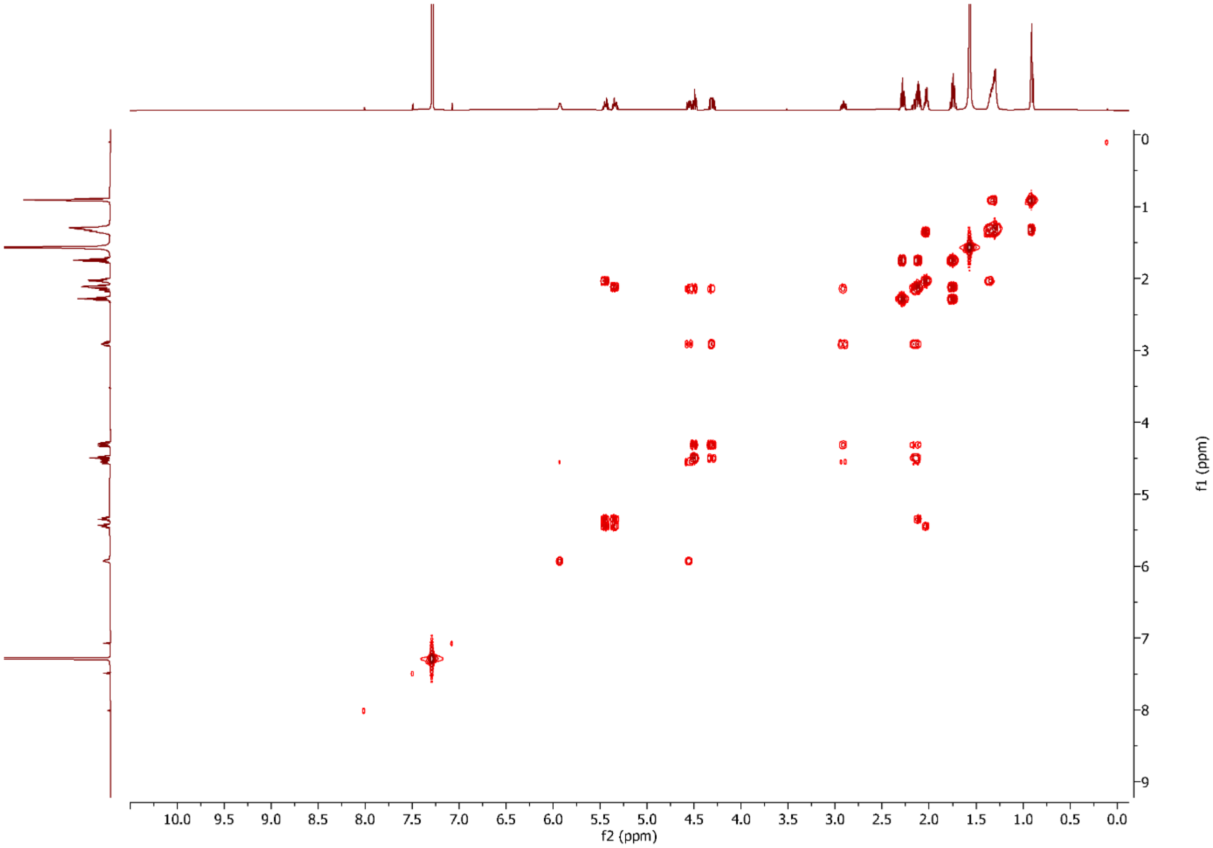

bioRxiv preprint doi: https://doi.org/10.64898/2026.07.02.736140; this version posted July 3, 2026. The copyright holder for this preprint (which was not certified by peer review) is the author/funder, who has granted bioRxiv a license to display the preprint in perpetuity. It is made available under a CC-BY 4.0 International license.

Figure S32

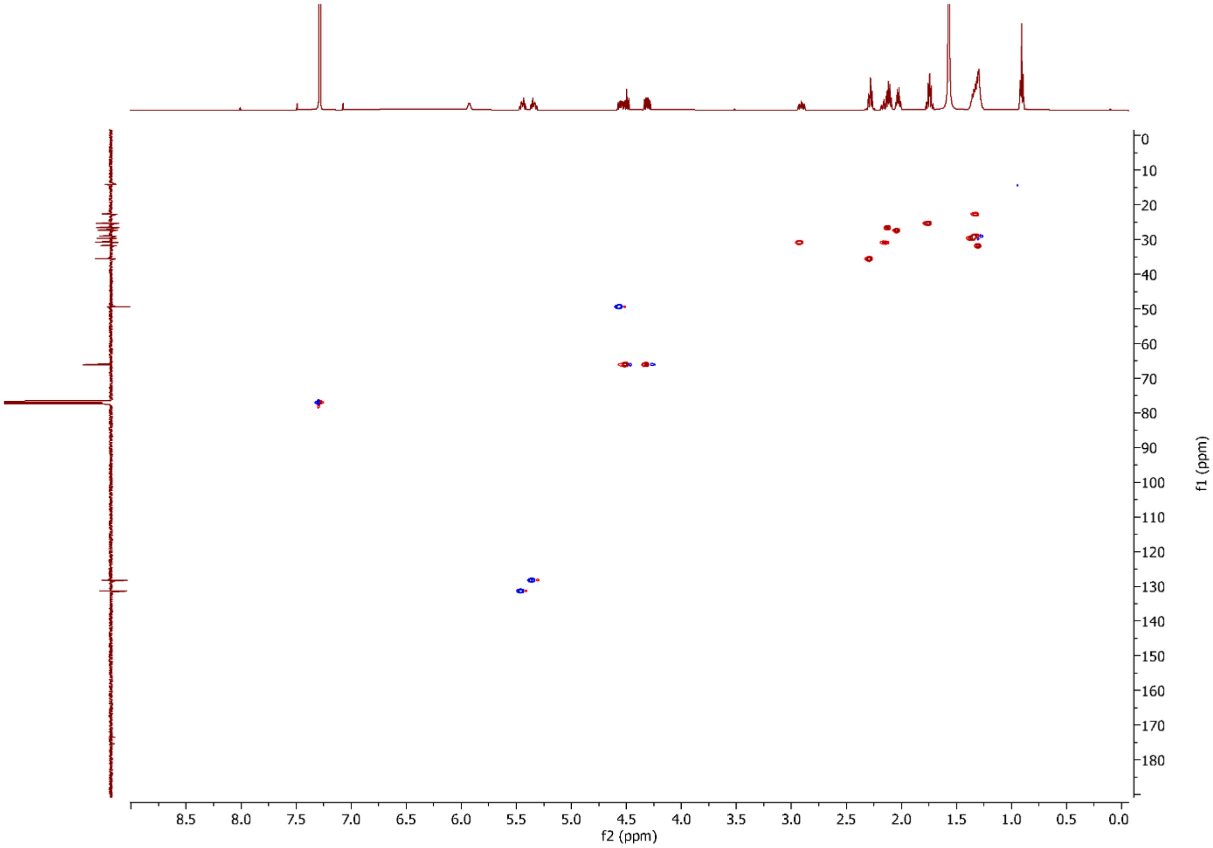

bioRxiv preprint doi: https://doi.org/10.64898/2026.07.02.736140; this version posted July 3, 2026. The copyright holder for this preprint (which was not certified by peer review) is the author/funder, who has granted bioRxiv a license to display the preprint in perpetuity. It is made available under a CC-BY 4.0 International license.

Figure S33

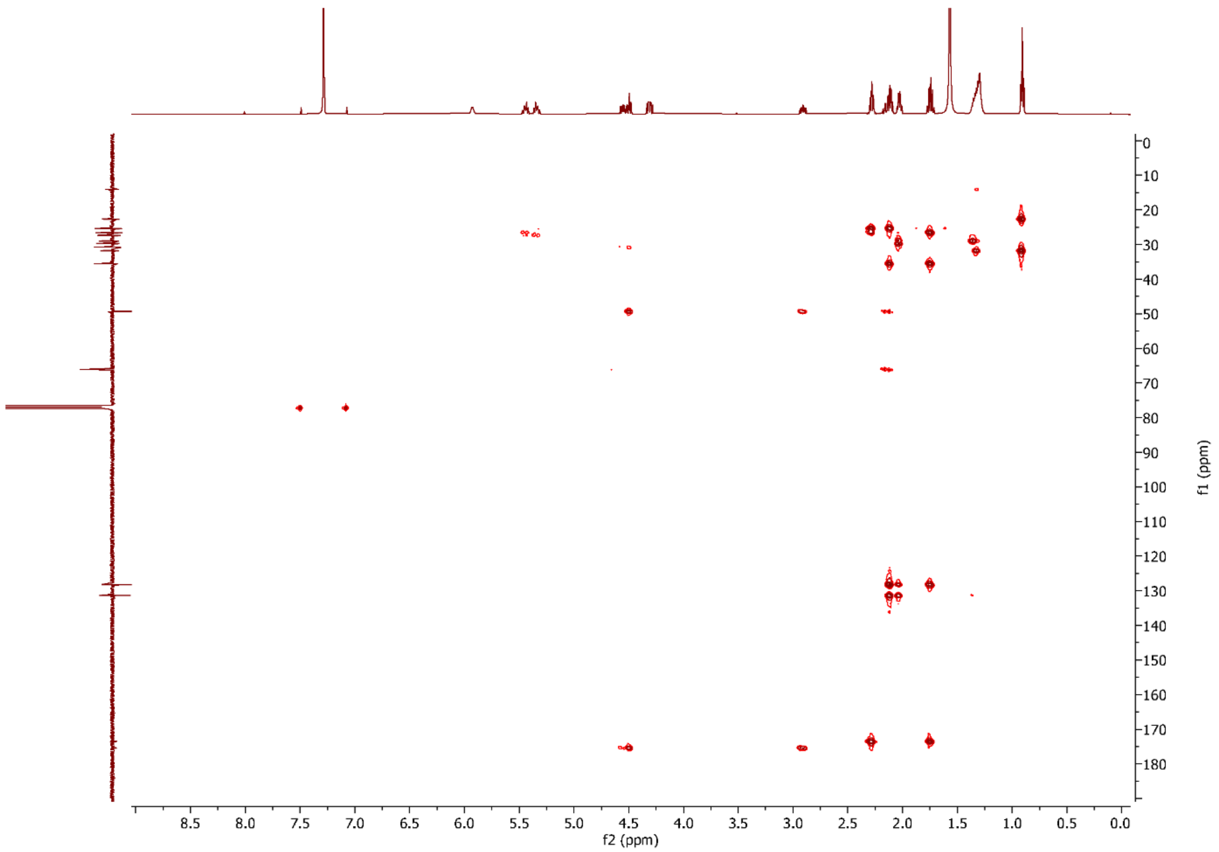

bioRxiv preprint doi: https://doi.org/10.64898/2026.07.02.736140; this version posted July 3, 2026. The copyright holder for this preprint (which was not certified by peer review) is the author/funder, who has granted bioRxiv a license to display the preprint in perpetuity. It is made available under a CC-BY 4.0 International license.

Figure S34

Reporter Strain
Δ*prophage A* Δ*prophage B*

Reporter Strain
Δ*prophage A* Δ*prophage B*
+
10 μM AI_{Host}

Reporter Strain
Δ*prophage A* Δ*prophage B*
+
Uninfected Host
Δ*prophage A* Δ*prophage B*

Reporter Strain
Δ*prophage A* Δ*prophage B*
+
Uninfected Host
Δ*prophage A* Δ*prophage B*
Δ*pgaI* Δ*pgaR*

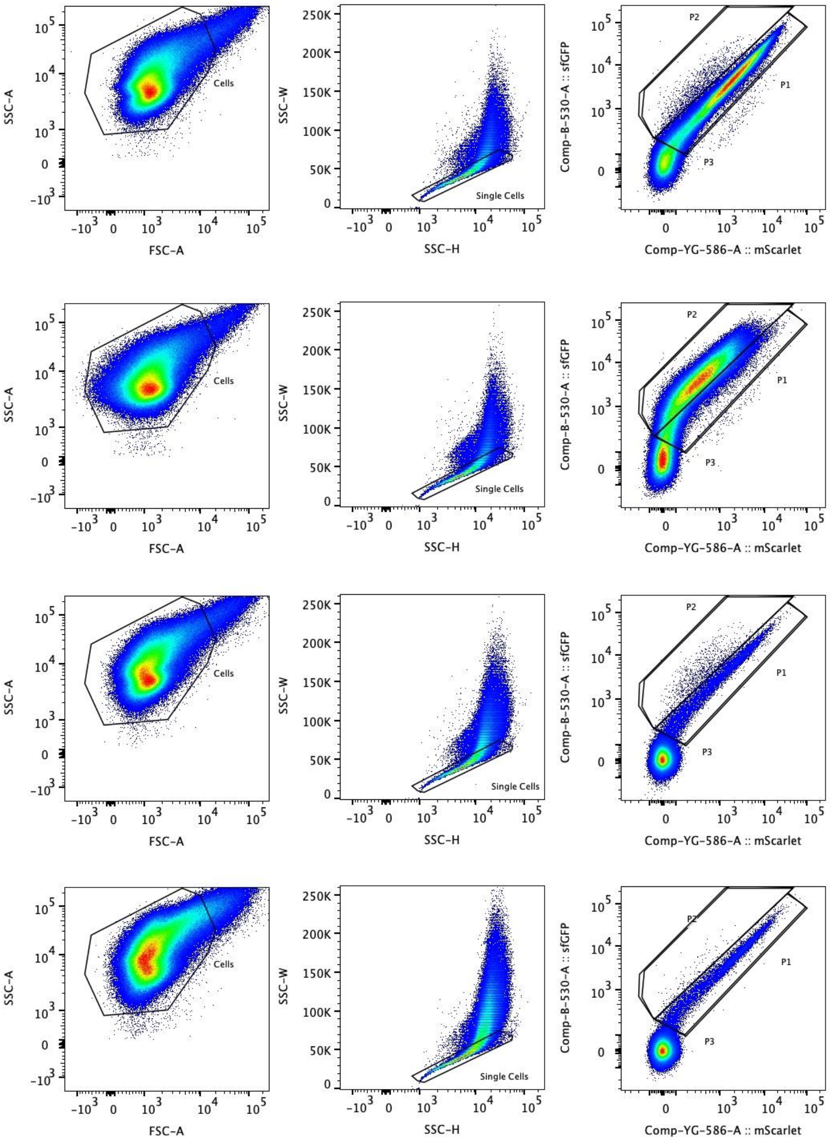

Supplement: Supplement 8 — Figure S1. Quantitation of and receptor dose responses to P. inhibens T5T HSLs. (A) Quantitation of HSLs produced by P. inhibens T5T and the related strain DSM17395 that lacks the QS prophages. Data were acquired from stationary phase cultures that had been grown for 16 h at 28°C with shaking at 200 rpm. (B) Dose response curves for host and prophage LuxR receptors to their cognate HSLs. The Host curve was generated from PpaaZ2-mScarlet-I in the P. inhibens Δ3luxI Δ3luxR strain carrying the pgaR gene. The Phage curves employed the respective PLysogeny–mScarlet-I-xre–luxR constructs in the P. inhibens Δ3luxI Δ3luxR strain. Strains were incubated with the indicated concentrations of HSLs and fluorescence was normalized to the culture OD600. Curves were fit using a four-parameter logistic model and the resulting EC50 values are indicated below each graph. Data represent means ± SDs from three (panel A) and four (panel B) biological replicates. n.d., not detected. Figure S2. Cross-activation of host and prophage QS circuits. (A) Responses of the host and prophage QS reporters to non-cognate HSLs. The Host data were generated from PpaaZ2-mScarlet-I in the P. inhibens Δ3luxI Δ3luxR strain carrying the pgaR gene. The Phage data were generated from the respective PLysogeny–mScarlet-I-xre–luxR–luxI constructs in the P. inhibens Δ3luxI Δ3luxR strain. Strains were grown in the presence of 10 μM of the indicated HSL and fluorescence was normalized to the OD600. (B) Dose responses of Host and Phage LuxR receptors to non-cognate HSLs. Dose-response curves for the indicated reporter and HSL. Curves were fit using a four-parameter logistic model and the resulting EC50 values are indicated below each graph. In both panels, data represent means ± SDs from four biological replicates. (C) Activity of the Phage B PLysogeny–mScarlet-I-xre-luxR-luxI reporter in the Δprophage A Δprophage B strain. Cultures were treated with DMSO (Mock) or 10 μM AIHost. Data represent means ± SDs from three [file NIHPP2026.07.02.736140v1-supplement-8.pdf]
